# Mechanical Characterisation of Single-Walled Carbon Nanotube Heterojunctions: Numerical Simulation Study

**DOI:** 10.3390/ma13225100

**Published:** 2020-11-12

**Authors:** André F. G. Pereira, Jorge M. Antunes, José V. Fernandes, Nataliya Sakharova

**Affiliations:** 1 Centre for Mechanical Engineering, Materials and Processes (CEMMPRE), Department of Mechanical Engineering, University of Coimbra, Rua Luís Reis Santos, Pinhal de Marrocos, 3030-788 Coimbra, Portugal; andre.pereira@dem.uc.pt (A.F.G.P.); jorge.antunes@dem.uc.pt (J.M.A.); valdemar.fernandes@dem.uc.pt (J.V.F.); 2 Polytechnic Institute of Tomar, Quinta do Contador, Estrada da Serra, 2300-313 Tomar, Portugal

**Keywords:** carbon nanotube heterojunctions, mechanical behaviour, rigidity, numerical simulations

## Abstract

The elastic properties of single-walled carbon nanotube heterojunctions were investigated using conventional tensile, bending and torsion tests. A three-dimensional finite element model was built in order to describe the elastic behaviour of cone heterojunctions (armchair–armchair and zigzag–zigzag). This comprehensive systematic study, to evaluate the tensile, bending and torsional rigidities of heterojunctions, enabled the formulation analytical methods for easy assessment of the elastic properties of heterojunctions using a wide range of their geometrical parameters.

## 1. Introduction

For more than two decades, one-dimensional (1D) nanomaterials, such as carbon nanotubes (CNTs), with unique mechanical, optical, thermal and electrical properties, have been a focus of research [1]. Afterwards, the research has included junctions of carbon nanotubes due to their prospective applications in nanodevices for electronics, biotechnology and health requests. Wei and Liu [2] published a detailed review describing the properties, manufacture, and potential applications of CNT junctions. Yengejeh et al. [3] reviewed the developments in modelling and numerical mechanical characterisation of structurally modified carbon nanotubes, paying special attention to CNT heterojunctions. 1D CNT heterojunctions are regarded as good candidates for functional elements in molecular electronics [4] and optics [5], and can be used as a building blocks for numerous nanoscale devices, including power-supply systems and functional thin-film electronics [6]. Among the many existing carbon nanotube junctions, such as those between two or more CNTs (for example, Y,T,X-shaped configurations), the multi-branched and ring-like junctions, the 1D heterojunctions with two endings, composed of single-walled carbon nanotubes (SWCNTs), have attracted significant research interest [2,7,8,9]. SWCNT heterojunctions (HJs), where two carbon nanotubes are seamlessly bonded together by a transition region, can originate metal-metal, metal-semiconductor or semiconductor-semiconductor intramolecular junctions, depending on diameter and chirality of the constituent SWCNTs. This makes SWCNT HJs suitable for nanoelectronic applications, such as rectifiers [9,10,11], diodes [12], quantum [13] and photovoltaic devices [14]. The fact that deformation of CNTs can significantly influence their electrical properties, changing their band structures and electrical conduction (for example, [15,16,17,18]) leads to other potential applications of SWCNT HJs, in electromechanical devices and as piezo-resistive strain sensors [17], and chemical and bio-sensors [19,20]. Regarding the numerous potential applications of the SWCNT HJs, it is of great importance to achieve their repeatable and controllable synthesis and large-scale fabrication. Various methods of synthesis of CNT HJs have been reported in the literature, among which are the chemical vapour deposition (CVD) process [8], the connection of individual CNTs by chemical reactions [21], electron beam [22] and current [23] welding, ion irradiation [24], and chemical doping [25]. Although numerous methods for producing CNT HJs have been established, it remains a challenge to produce heterojunctions in industrial scale, accompanied by the fast characterisation of their structure. Recently, An et al. [4] suggested an efficient method for the controllable preparation of SWCNT HJs by means of growth of ultra-long SWCNTs and their subsequent modifying to create HJ regions, on trenched substrate. In addition to the rapid identification of the heterojunctions produced, this method establishes how to manufacture the SWCNT HJs with the required structure in large quantities.

In spite of the latest advances accomplished in this research domain, various questions still exist. As the strength and productivity of the nanodevices depend on the mechanical properties of their constituents, one of these remaining problems is to correctly describe the deformation behaviour of CNT heterojunctions.

Melchor and Dobrado [26] outlined two foremost heterojunction configurations: (i) cone-HJs (heterojunctions of two nanotubes having the same chiral angle, but different radii), such as armchair–armchair and zigzag–zigzag heterojunctions, and (ii) radius-preserving HJs, constituting by two nanotubes having different chiral angles, such as armchair–zigzag or chiral–armchair (or zigzag) heterojunctions. Ghavamian et al. [27] designate the cone-heterojunctions by HJs with straight connection, while the heterojunctions that preserve the radius are called HJs with bent connection. Yao et al. [8] pointed out the fact that the majority of HJs (>95%) are cone-heterojunctions.

The molecular dynamics (MD) and nanoscale continuum modelling (NCM) approaches, later accompanied by finite element (FE) modelling, were established as predominant methods to simulate the mechanical behaviour of CNT heterojunctions. Several works employing the MD approach [28,29,30] were carried out. Lee and Su [28] used an MD simulation approach with reactive empirical bond-order (REBO) potential for describing the interaction between carbon atoms in their study of the effect of temperature on yield stress and Young’s modulus of single-walled carbon nanotube (SWCNT) HJs under tension and compression. Li et al. [29] employed a REBO potential to investigate the influence of temperature and strain rate on the tensile and failure behaviour of single-walled and double-walled CNT HJs.

Qin et al. [30] published a study using MD simulation with second-generation Tersoff–Brenner potential to assess the Young’s modulus and failure stress of single- and double-walled CNT HJs. Xi et al. [31] used the same potential in their MD study on the mechanical behaviour of HJs structures containing four (n,n) armchair and five (2n,0) zigzag SWCNTs. Kang et al. [32] studied the HJs buckling behaviour under compression, also resorting to the MD simulation with Tersoff–Brenner potential coupled with NCM approach. Kinoshita et al. [33] calculated the Young’s modulus of (8, 0)–(6, 0)–(8, 0) SWCNT HJ structures using ab initio density functional theory calculations.

The elastic properties of HJs have been evaluated under the tensile [34,35,36,37,38] and torsion [27,35,39,40,41] loading conditions, using the NCM approach. Ghavamian et al. and Yengejeh et al. [35,42] used the NCM approach for studying the buckling behaviour of HJs. Sakharova et al. [39], assessing the mechanical properties of the armchair–armchair and zigzag–zigzag HJs, noticed redundant bending deformation occurs under tension, which complicates the analysis of the results of the tensile test. Scarpa et al. [34] also described this feature when analysing the tensile test results of (5, 5)–(10, 10) HJs, in order to calculate the Poisson’s ratio. Most of the studies on the evaluation of the HJ elastic properties are dealing with cone-heterojunctions [30,34,36,38,39,40,41], and only a few works described the mechanical behaviour of the HJs with bent connection [27,35]. The common finding of some of these works [27,35,36,38,39,40] is the reduction of the elastic properties of the HJs (their rigidities, Young’s and shear moduli), when compared to the elastic properties of the constituent SWCNTs. The results of aforementioned works point out that a comprehensive systematic study is required for better understanding the mechanical behaviour of HJs.

The present study aims to provide new results to accomplish the modelling of armchair–armchair and zigzag–zigzag SWCNT heterojunctions and the systematic characterisation of their mechanical behaviour, resorting to the NCM approach coupled with the three dimensional (3D) FE method. In this context, a systematic parametric study on the tensile, bending and torsional rigidities of SWCNT HJs was carried out. A robust methodology is recommended that allows assessing the three rigidities of HJs in a wide range of their average diameters and differences between diameters of constituent SWCNTs, without recourse to numerical simulation.

## 2. Materials and Methods

### 2.1. Geometric Definition of SWCNT HJs

**Figure 1:**
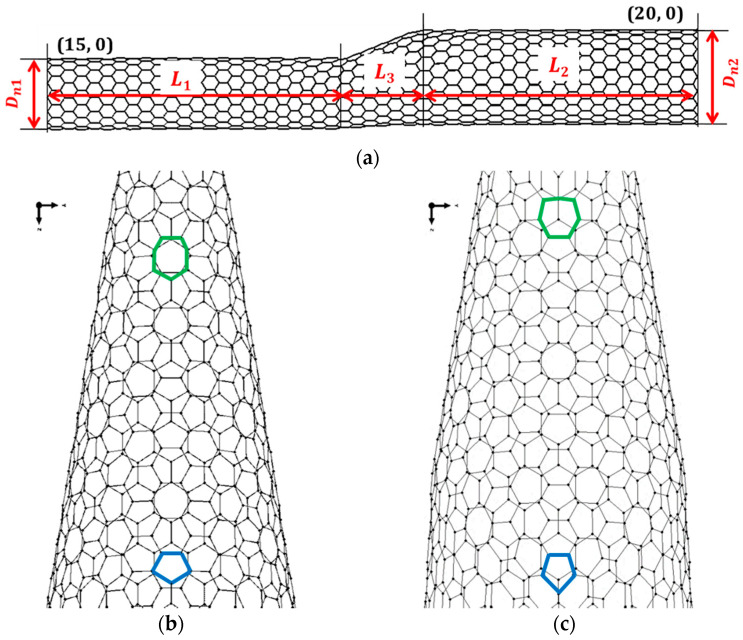
(**a**) Geometry of the cone zigzag–zigzag (15, 0)–(20, 0) HJ. (**b**,**c**) Heptagon (in bold green) and pentagon (in bold blue) defects in the intermediate region of (**b**) armchair–armchair (5, 5)–(15, 15) and (**c**) zigzag–zigzag (15, 0)–(25, 0) HJs. HJ structures are obtained using the academic software CoNTub 1.0© [26].

The SWCNT heterojunction structure can be seen as two SWCNTs coupled by an intermediate region, as shown in Figure 1a, for the case of cone-heterojunction. The intermediate region contains Stone–Wales defects [26], as shown in Figure 1b,c (heptagon in bold green and pentagon in bold blue).

The overall length of the cone-heterojunction is expressed by:

(1) $$L_{\mathrm{HJ}}=L_{1}+L_{2}+L_{3}$$

where *L*_1_, *L*_2_ are the lengths of the HJs in the narrow and wide SWCNT regions, respectively, and *L*_3_ is the length of the connecting region (Figure 1a).

When cone-heterojunction is considered (i.e., the heterojunction is composed by two SWCNTs with different diameters), the diameter of HJ can be presented as the average of the narrow (*D*_*n*1_), and wide (*D*_*n*2_) diameters (for example [26]):

(2) $${\bar{D}}_{\mathrm{HJ}}=\frac{1}{2}(D_{n1}+D_{n2})$$

The aspect ratio of the cone-heterojunction (armchair–armchair and zigzag–zigzag HJs) is defined as [29]:

(3) $$\eta =\frac{L_{3}}{{\bar{D}}_{\mathrm{HJ}}}$$

**Figure 2:**
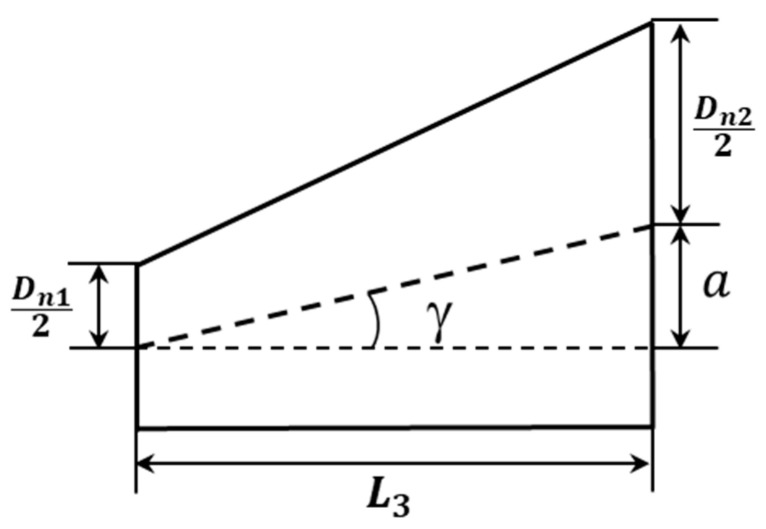
Connecting region of the single-walled carbon nanotube (SWCNT) HJ.

According to a previous study by the authors [39] for the armchair–armchair and zigzag–zigzag HJs, the angle *γ* between the direction of the axis of the SWCNTs, constituting the HJs, and the junction centre line as shown in Figure 2, is equal to 12.7°, whatever the diameters of constituent SWCNTs, and the length of the connecting region, *L*_3_, is a quasi linear function of (*D*_*n*2_ − *D*_*n*1_). The fitted straight line equation for determination of *L*_3_ is as follows:

(4) $$L_{3}=\left(D_{n2}{-D}_{n1}\right)$$

Based on geometrical analysis, a similar expression for the length of the connecting region, *L*_3_, of cone-heterojunctions was suggested by Qin et al. [30]:

(5) $$L_{3}=\frac{\sqrt{3}}{2}\pi \left(D_{n2}{-D}_{n1}\right)=2.7207\left(D_{n2}-D_{n1}\right)$$

### 2.2. FE Modelling

For modelling of SWCNT HJs, the NCM approach, which is based on the replacement of the C–C bonds by the equivalent beam elements, was employed. The bases for the application of continuum mechanics in order to describe the mechanical behaviour of CNT HJs are the established relations between inter-atomic potential energies of the molecular structure and strain energies of the equivalent continuum structure comprised of elastic beams, undergoing axial, bending and torsional deformations [35,38]. The input values for the FE model are given in Table 1.

**Figure 3:**
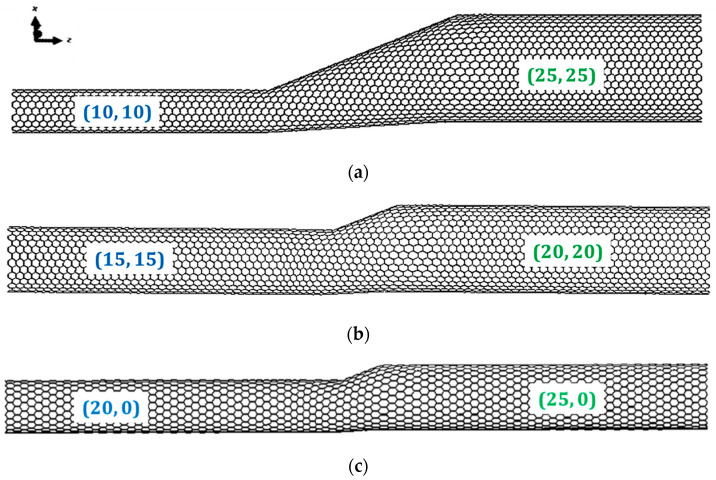
FE meshes of HJs: (**a**) armchair–armchair (10, 10)–(25, 25); (**b**) armchair–armchair (15, 15)–(20, 20) and (**c**) zigzag–zigzag (20, 0)–(25, 0); structures obtained using the academic software CoNTub 1.0 [26].

The meshes of the SWCNT HJ structures, used in the FE analyses, were constructed using the CoNTub 1.0 software (University of Granada, Granada, Spain) [26]. This code generates American Standard Code for Information Interchange (ASCII) files, describing atom positions, which can be entered as input in the finite element analysis (FEA) code, in order to perform the simulation of mechanical tests. To convert the ASCII files, obtained using the CoNTub 1.0 program, into the format usable by the commercial FEA code ABAQUS^®^ (Abaqus 2020, HKS Inc., East Providence, RI, USA, an in-house application previously developed, designated InterfaceNanotubes [45], was used. Examples of FE meshes for SWCNT HJs, armchair–armchair and zigzag–zigzag, are shown in Figure 3.

The geometrical characteristics of SWCNT HJs used in present FE analyses are shown in Table 2. In a previous study [39], the influence of the overall length of HJ structures on their bending and torsional rigidities was examined for heterojunctions made up of constituent nanotubes of equal length and having one, two and three orders of magnitude the length of the connecting junction region. Based on these results, the SWCNTs HJs of the present work were built up in such way that the length of the constituent SWCNTs is about two orders of magnitude of the length of the connecting junction region.

### 2.3. Loading Conditions

**Figure 4:**
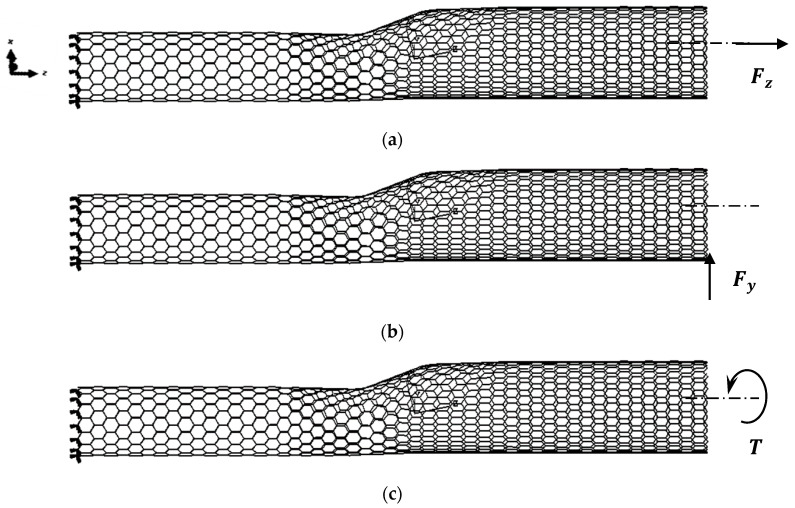
Loading and boundary conditions for zigzag–zigzag (15, 0)–(20, 0) HJ, for the following tests: (**a**) tensile; (**b**) bending; (**c**) torsional.

Conventional numerical tensile, bending and torsional tests were used in order to evaluate the respective rigidities of the SWCNT HJs. The applied loading and boundary conditions are explained in Figure 4. Two loading conditions, which include fixing the narrow and the wide sides of the HJ structure, were considered in the tensile, bending and torsion tests.

The tensile rigidity, (*EA*)*_HJ_*, of SWCNT HJ structures is determined as follows:

(6) $${\left(\mathrm{EA}\right)}_{\mathrm{HJ}}=\frac{F_{z}L_{\mathrm{HJ}}}{u_{z}}$$

where *F_z_* is the axial tensile force applied at one end of the heterojunction, leaving the other end fixed, *L_HJ_* is the heterojunction length and *u_z_* is the axial displacement obtained from the FE analysis.

The bending rigidity, (*EI*)*_HJ_*, is determined as:

(7) $${\left(\mathrm{EI}\right)}_{\mathrm{HJ}}=\frac{F_{y}{L_{\mathrm{HJ}}}^{3}}{{3u}_{y}}$$

where *F_y_* is the transverse force applied at one end of the heterojunction, leaving the other fixed, *u_y_* is the transverse displacement, obtained from the FE analysis.

The torsional rigidity, (*GJ*)*_HJ_*, is determined by:

(8) $${\left(\mathrm{GJ}\right)}_{\mathrm{HJ}}=\frac{{\mathrm{TL}}_{\mathrm{HJ}}}{\varphi }$$

where T is torsional moment applied at one end of the heterojunctions, leaving the other fixed and *φ* is the twist angle, obtained from the FE analysis. The nodes at the end of the heterojunction, on which the load is applied, are inhibited from moving in the radial direction.

## 3. Results and Discussion

### 3.1. Rigidities of SWCNT Heterojunctions

#### 3.1.1. Parametric Study of Rigidities of SWCNT Heterojunctions: FE Analysis

The values of the tensile (*EA*)*_HJ_*, bending (*EI*)*_HJ_* and torsional (*GJ*)*_HJ_* rigidities were calculated by Equations (6)–(8), respectively, using the data taken from the FE analysis. The effect of the difference between the wide and the narrow diameters of the nanotubes of each HJ, ∆*D* = *D*_*n*2_ − *D*_*n*1_, the heterojunction aspect ratio, η, and the average HJ diameter, ${\bar{D}}_{\mathrm{HJ}}$, was studied in all three rigidities.

**Figure 5:**
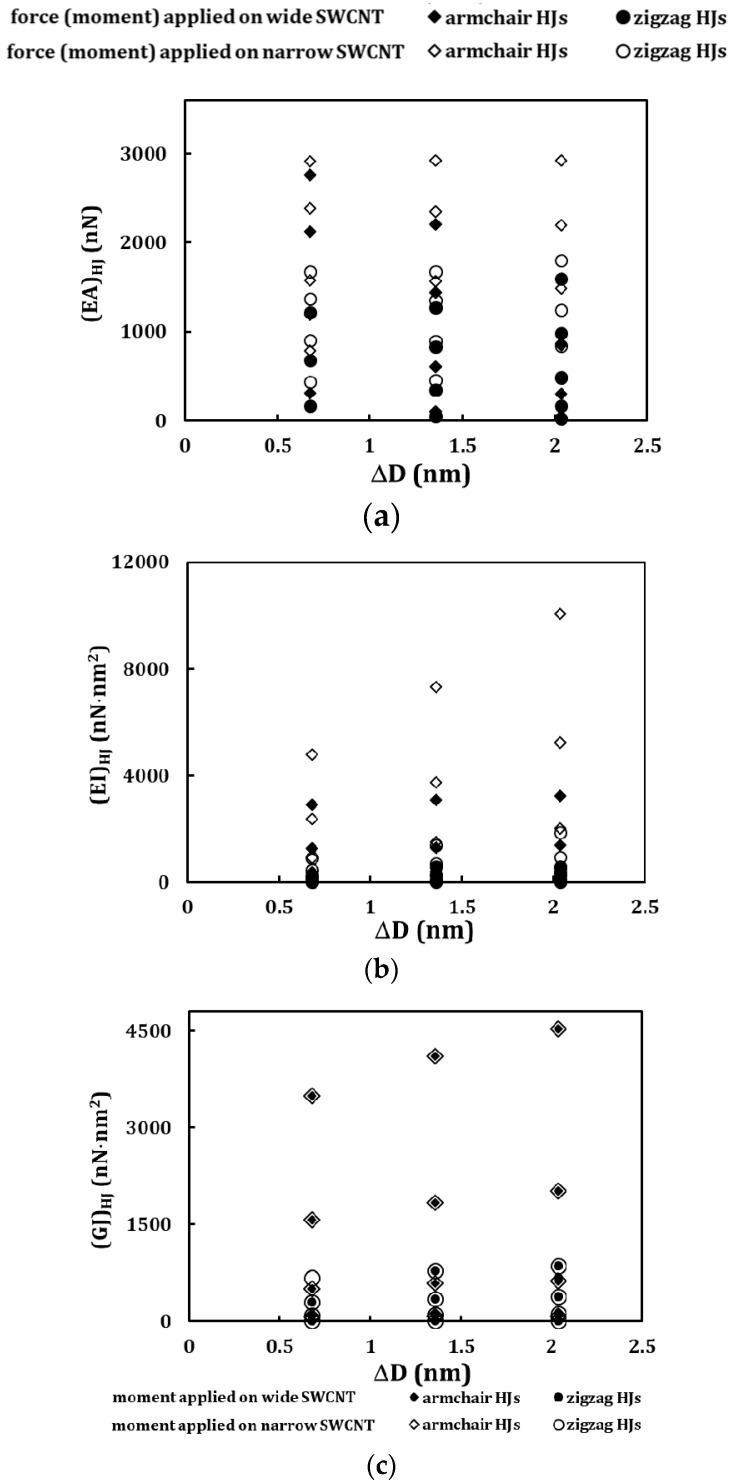
Evolution of the rigidities of the HJs with the difference between the narrow and wide nanotubes, ∆*D*, for armchair–armchair and zigzag–zigzag HJs: (**a**) (*EA*)*_HJ_*, (**b**) (*EI*)*_HJ_* and (**c**) (*GJ*)*_HJ_*.

The tensile (*EA*)*_HJ_*, bending (*EI*)*_HJ_* and torsional (*GJ*)*_HJ_* rigidities for armchair–armchair and zigzag–zigzag HJs were plotted in Figure 5 as a function of the difference between diameters of the wide and narrow nanotubes, ∆*D*. The values obtained for the two loading conditions are shown. The values of the (*EA*)*_HJ_* rigidities are grouped by the three values of ∆*D* considered, with the higher (*EA*)*_HJ_* values corresponding to lower heterojunction aspect ratios, $\eta =L_{3}/{\bar{D}}_{\mathrm{HJ}}$. The same behaviour is observed for the (*EI*)*_HJ_* and (*GJ*)*_HJ_* rigidities.

**Figure 6:**
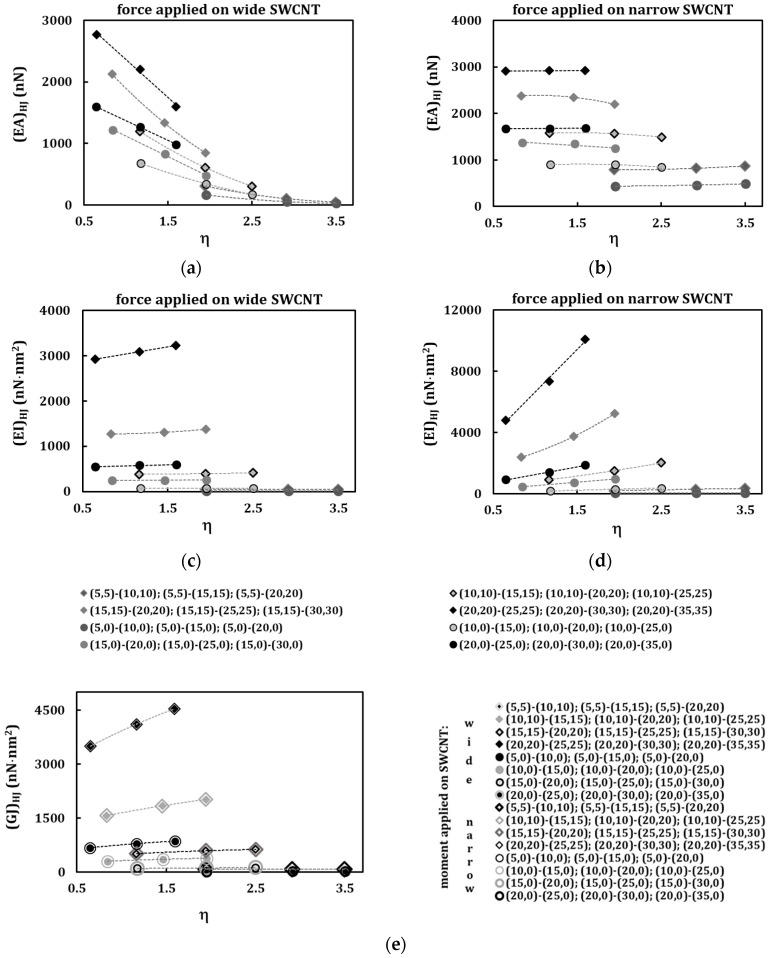
(**a**–**d**) Evolution of (**a**,**b**) tensile (*EA*)*_HJ_*, (**c**,**d**) (*EI*)*_HJ_* rigidities, with the aspect ratio, *η*, for armchair–armchair and zigzag–zigzag HJs; the force is applied to the wide SWCNT at (**a**,**c**) and to the narrow SWCNT at (**b**,**d**). (**e**) Evolution of torsional, (*GJ*)*_HJ_*, rigidity with the aspect ratio, *η*; the moment applied to the narrow and to the wide side of the HJ structure.

To complement the presentation of the HJ rigidity values, the results of Figure 5 were plotted as a function of the heterojunction aspect ratio, *η* (Figure 6a–e). The results are grouped (see lines in the figures) so that the narrow nanotube is the same in each group. Two loading conditions were considered, with the force (or moment) being applied to the wide and narrow nanotube.

The tensile rigidity, (*EA*)*_HJ_*, decreases with the increase of the heterojunction aspect ratio, *η*, when the force is applied on the wide SWCNT, for armchair and zigzag HJs (Figure 6a). When the force is applied in the narrow SWCNT, the (*EA*)*_HJ_* rigidity is nearly constant with increasing of heterojunction aspect ratio, *η* (Figure 6b). The (*EA*)*_HJ_* values for armchair–armchair HJs are, in most cases, higher than those for zigzag–zigzag HJs.

The bending rigidity, (*EI*)*_HJ_*, shows non-significant increase with the increase in HJ aspect ratio, η, when the force is applied on the wide SWCNT (Figure 6c). This increase in (*EI*)*_HJ_* with the HJ aspect ratio can be considerable when the force is applied to the narrow SWNCT (Figure 6d). For both loading conditions, the (*EI*)*_HJ_* values remain almost constant with η for the HJ sequences (5, 5)–(10, 10), (5, 5)–(15, 15), (5, 5)–(20, 20) and (5, 0)–(10, 0), (5, 0)–(15, 0), (5, 0)–(20, 0), i.e., those with smaller narrow SWCNTs.

The evolution of the torsional rigidity with the HJ aspect ratio, *η* is insensitive to the loading condition: the (*GJ*)*_HJ_* values are approximately identical whether the torsional moment is applied to the wide or narrow nanotube, and (*GJ*)*_HJ_* rigidities for armchair–armchair HJs are, in most cases, higher than those for zigzag–zigzag HJs (Figure 6e). The torsional rigidity, (*GJ*)*_HJ_*, increases with the increase of the HJ aspect ratio, *η*, and this increase is less significant for HJ groups with smaller narrow SWCNT.

**Figure 7:**
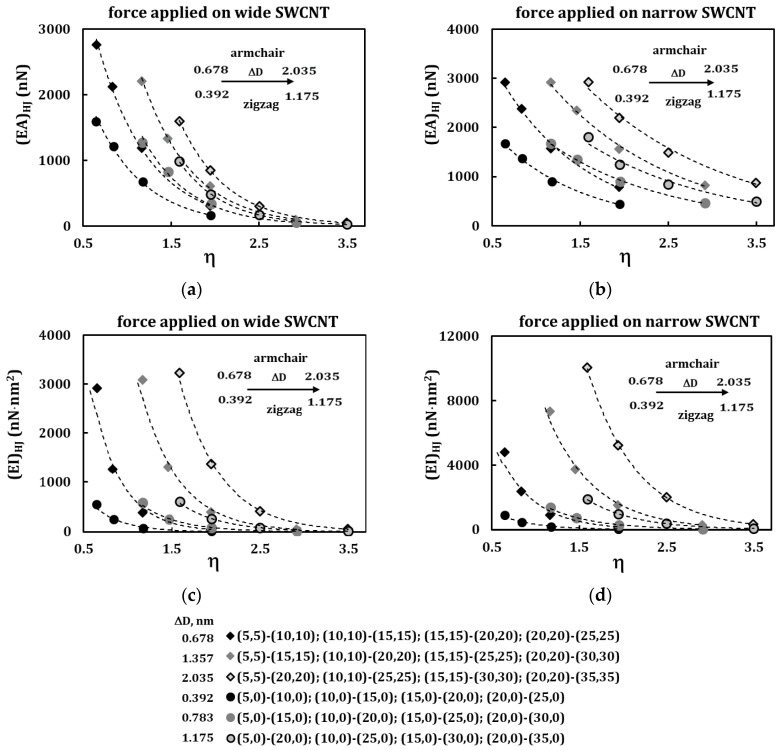

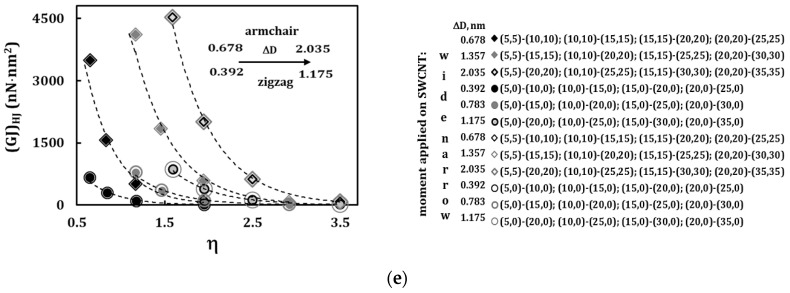
(**a**–**d**) Evolution of the (**a**,**b**) tensile, (*EA*)*_HJ_*, (**c**,**d**) bending, (*EI*)*_HJ_*, rigidities with the HJ aspect ratio, η; the force is applied to the wide SWCNT at (**a**,**c**) and to the narrow SWCNT at (**b**,**d**). (**e**) Evolution of the torsional, (*GJ*)*_HJ_*, rigidity with the HJ aspect ratio, η.

The results of Figure 6 can be represented as shown in the Figure 7 for the tensile (*EA*)*_HJ_*, bending (*EI*)*_HJ_* and torsional (*GJ*)*_HJ_* rigidities as a function of the HJ aspect ratio, *η*, for the both loading conditions. This figure shows heterojunction sequences that have the same difference between the diameters of the wide and narrow nanotubes, ∆*D* = *D*_*n*2_ − *D*_*n*1_, i.e., ∆*D* = 0.678, 1.357, 2.035 nm for armchair–armchair HJs, ∆*D* = 0.392, 0.783, 1.175 for zigzag–zigzag HJs. The evolutions of the tensile (*EA*)*_HJ_* rigidity follow exponential trend, regardless of whether the force is applied to the wide or narrow nanotube (Figure 7a,b). The same type of trend occurs for the evolutions of the bending (*EI*)*_HJ_* and torsional (*GJ*)*_HJ_* rigidities with *η* (Figure 7c–e).

**Figure 8:**
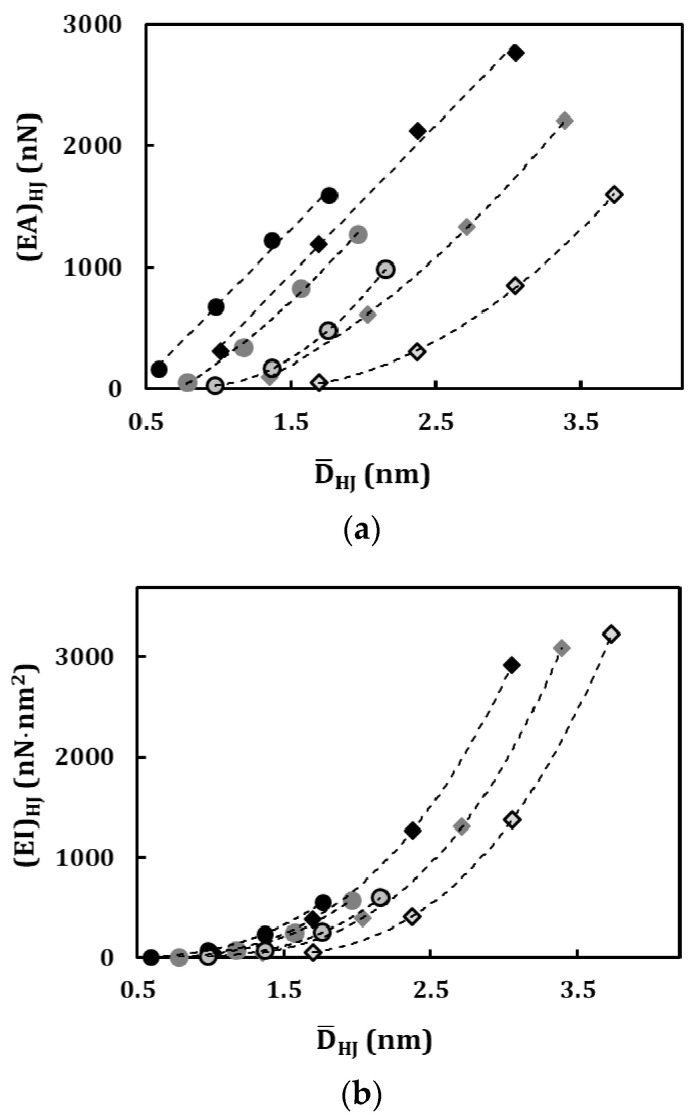

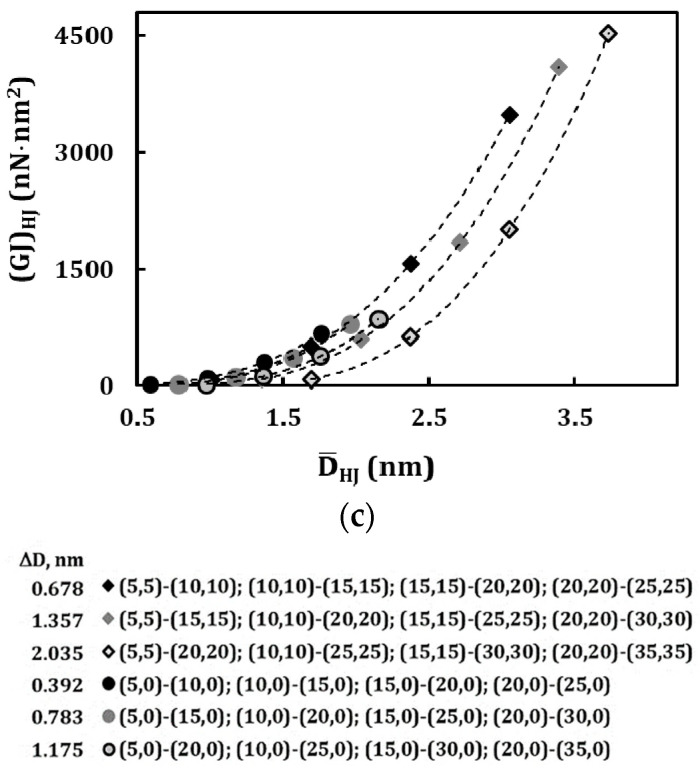
Evolution of the (**a**) tensile (*EA*)*_HJ_*, (**b**) bending, (*EI*)*_HJ_*, and (**c**) torsional (*GJ*)*_HJ_*, rigidities with the average HJ diameter ${\bar{D}}_{\mathrm{HJ}}$. The force (or moment) is applied to the wide SWCNT.

In Figure 8, the evolutions of the (*EA*)*_HJ_*, (*EI*)*_HJ_* and (*GJ*)*_HJ_* rigidities of armchair–armchair and zigzag–zigzag HJs are plotted as a function of the average HJ diameter ${\bar{D}}_{\mathrm{HJ}}=\frac{1}{2}(D_{n1}+D_{n2})$ for the loading condition in which the force (moment) is applied on the wide nanotube. All three rigidities increase with the increase of the average HJ diameter. The (*EA*)*_HJ_* evolutions can be separated for armchair–armchair and zigzag–zigzag HJs. This is also true for (*EI*)*_HJ_* and (*GJ*)*_HJ_* evolutions. The evolutions of the (*EA*)*_HJ_* rigidity follow quasi-linear trend for heterojunctions with ∆*D* = 0.678 nm (armchair–armchair) and ∆*D* = 0.392 nm (zigzag–zigzag). For bigger ∆*D*, equal to 1.357 and 2.035 nm for armchair–armchair HJs and equal to 0.783 and 1.175 nm for zigzag–zigzag HJs, the (*EA*)*_HJ_* evolutions are close to a second degree polynomial trend. It was previously shown [45] that the evolution of (*EA*)*_HJ_* rigidity of SWCNT with its diameter follows a quasi-linear trend. The linear dependence between the (*EA*)*_HJ_* rigidity and the average HJ diameter, for small values of ∆*D*, reveals that the HJ structure behaves like a homogeneous SWCNT under tensile loading test. However, for higher ∆*D* values, when the (*EA*)*_HJ_* evolutions deviate from the linear trend and become close to a second degree polynomial trend, this difference can most likely be attributed to the redundant bending deformation, observed during the tensile test of these HJs [39]. It influences the tensile rigidity results of HJs in which the difference between diameters of the wide and narrow nanotubes, ∆*D*, are greater than 0.678 nm and 0.392 nm, for armchair–armchair and zigzag–zigzag HJs, respectively. The evolutions of the bending, (*EI*)*_HJ_*, and torsional, (*GJ*)*_HJ_*, rigidities with the average HJ diameter ${\bar{D}}_{\mathrm{HJ}}$ are close a third degree polynomial trend.

#### 3.1.2. Rigidities of SWCNT Heterojunctions: Analytical Solution

The tensile, (*EA*)*_HJ_*, bending, (*EI*)*_HJ_*, and torsional, (*GJ*)*_HJ_*, rigidities of the heterojunctions can be calculated from the rigidities of the constituent SWCNTs. For this, a structural analysis of an equivalent structure composed of three beams, representing the two SWCNTs and the connection region, was performed assuming that the length of this region is much smaller than that of the SWCNTs. Based on this analysis, the (*EA*)*_HJ_* rigidity is given by

(9) $${\left(\mathrm{EA}\right)}_{\mathrm{HJ}}=\frac{L_{\mathrm{HJ}}}{\left(\frac{L_{a}}{{\left(\mathrm{EA}\right)}_{a}}+\frac{a^{2}L_{f}}{{\left(\mathrm{EI}\right)}_{f}}+\frac{L_{f}}{{\left(\mathrm{EA}\right)}_{f}}\right)}$$

where *L_HJ_* is the overall length of HJ; *a* is a geometrical parameter described in Figure 3; (*EA*)*_a_* and (*EA*)*_f_* are the tensile rigidities of the SWCNTs that comprise HJ and (*EI*)*_f_* is the corresponding bending rigidity; *L_a_* and *L_f_* are the lengths of the constituent SWCNTs; the subscript *a* refers to the nanotube on which the force (moment) is applied and the subscript *f* refers to the fixed nanotube.

Based on similar structural analyses, it is possible to obtain (*EI*)*_HJ_* and (*GJ*)*_HJ_* rigidities, respectively, as already suggested in a preliminary analysis [40]:

(10) $${\left(\mathrm{EI}\right)}_{\mathrm{HJ}}=\frac{L_{\mathrm{HJ}}^{3}}{\left(\frac{L_{a}^{3}}{{\left(\mathrm{EI}\right)}_{a}}+\frac{{3L}_{a}^{2}L_{f}{+3L}_{a}L_{f}^{2}{+L}_{f}^{3}}{{\left(\mathrm{EI}\right)}_{f}}\right)}$$

(11) $${\left(\mathrm{GJ}\right)}_{\mathrm{HJ}}=\frac{L_{\mathrm{HJ}}}{\left(\frac{L_{a}}{{\left(\mathrm{GJ}\right)}_{a}}+\frac{L_{f}}{{\left(\mathrm{GJ}\right)}_{f}}\right)}$$

where (*EI*)*_a_* and (*EI*)*_f_* are the bending rigidities of the SWCNTs comprising HJ and (*GJ*)*_a_* and (*GJ*)*_f_* are their torsional rigidities.

The rigidities of the individual SWCNTs, which constitute the HJ structures, can be taken directly from FE analysis or evaluated analytically as follows [40,45]:

(12) $${\mathrm{EA}}_{\mathrm{SW}}=\alpha_{\mathrm{SW}}\left(D_{n}-D_{0}\right)$$

(13) $${\mathrm{EI}}_{\mathrm{SW}}=\beta_{\mathrm{SW}}{\left(D_{n}{-D}_{0}\right)}^{3}$$

(14) $${\mathrm{GJ}}_{\mathrm{SW}}=\gamma_{\mathrm{SW}}{\left(D_{n}-D_{0}\right)}^{3}$$

where *D_n_* is the SWCNT diameter and $\alpha_{\mathrm{SW}}$, $\beta_{\mathrm{SW}}$, $\gamma_{\mathrm{SW}}$ and *D*_0_ are fitting parameters. The values of the fitting parameters are: $\alpha_{\mathrm{SW}}=1121.20\mathrm{nN}/\mathrm{nm}$, $\beta_{\mathrm{SW}}=140.25\mathrm{nN}/\mathrm{nm}$, $\gamma_{\mathrm{SW}}=130.39\mathrm{nN}/\mathrm{nm}$ and *D*_0_ is considered equal to zero, as its value is negligible when compared with *D_n_* [40].

**Figure 9:**
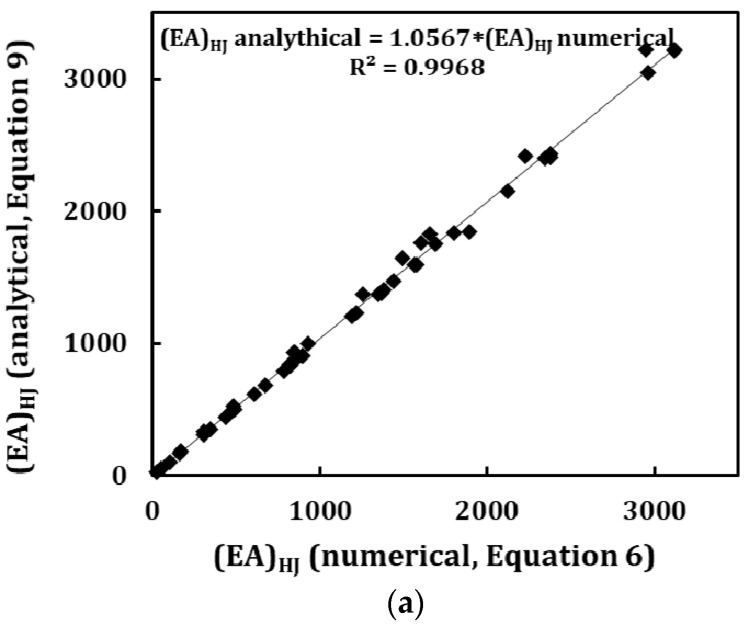

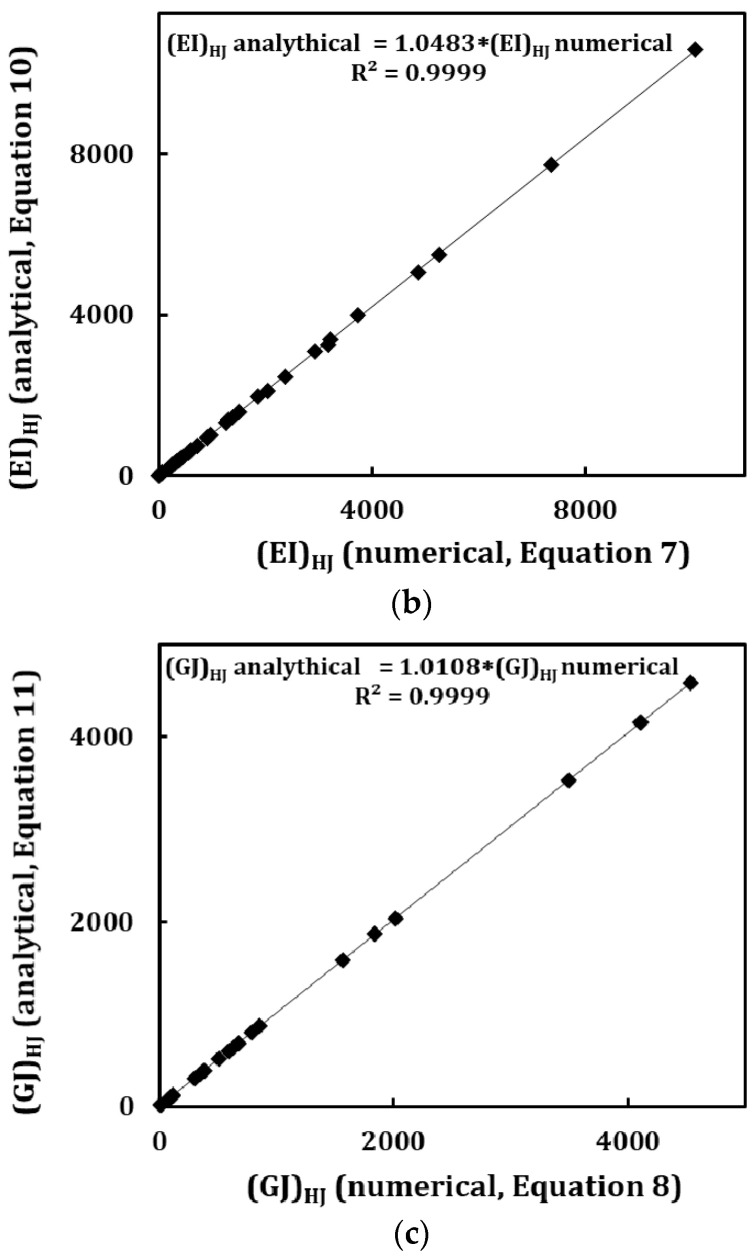
Comparison between the (**a**) tensile, (*EA*)*_HJ_*, (**b**) bending, ${\left(\mathrm{EI}\right)}_{\mathrm{HJ}}$ and (**c**) torsional, ${\left(\mathrm{GJ}\right)}_{\mathrm{HJ}}$ rigidities obtained from the FE analysis (Equations (6)–(8)) and those from Equations (9)–(11) for armchair–armchair and zigzag–zigzag HJs. Both loading conditions, with the force (or moment) applied to the narrow and to the wide side of the HJ structure, are considered.

Figure 9 compares the values of the three rigidities obtained from FE analysis (Equations (6)–(8)) and those calculated by Equations (9)–(11). In these last equations, the input values of *EA_SW_*, *EI_SW_* and *GJ_SW_* for individual constituent SWCNTs were obtained from FE analysis. As an alternative these rigidity values can be assessed from Equations (12)–(14) which allow accurate evaluation of the SWCNTs rigidities [40,45]. The results of Figure 9 reveal the accurateness of the proposed analytical expressions for evaluation of the tensile, bending and torsional rigidities of armchair–armchair and zigzag–zigzag HJs. The fitted linear equations show that the average difference between the rigidity values obtained from FE analysis and those calculated analytically are 5.67%, 4.33% and 1.08% for the (*EA*)*_HJ_*, (*EI*)*_HJ_* and (*GJ*)*_HJ_* rigidities, respectively. In addition to Figure 9, the values of the (*EA*)*_HJ_*, (*EI*)*_HJ_* and (*GJ*)*_HJ_* rigidities obtained from FE analysis (Equations (6)–(8)) and those calculated by Equations (9)–(11) are shown in Appendix A (Table A1). The greatest differences (about 10%), occur for the tensile rigidity, (*EA*)*_HJ_*, and are observed in HJs which have simultaneously large mean diameter, ${\bar{D}}_{\mathrm{HJ}}$, and large differences, ∆*D*, between the diameters of the wide and narrow nanotubes, for which redundant bending deformation in tension is observed.

#### 3.1.3. Analytical Study for Evaluation of the Rigidities of SWCNT Heterojunctions

In order to develop an analytical procedure for assessing the HJ rigidities, in addition to the HJ structures in Table 2, heterojunctions with the geometrical characteristics, shown in Table 3 (armchair–armchair HJs) and Table 4 (zigzag–zigzag HJs), were also considered. These HJ structures are organized by sequences defined as $\left(n_{1}^{i}{,m}_{1}^{i}\right){–(n}_{2}^{i}{=n}_{1}^{i}{+C,m}_{2}^{i}{=m}_{1}^{i}+C)$, for armchair–armchair HJs, and $\left(n_{1}^{i},0\right){–(n}_{2}^{i}{=n}_{1}^{i}+C,0)$, for zigzag–zigzag HJs, where C is the difference between the chiral indices of the narrow $\left(n_{1}^{i}{,m}_{1}^{i}\right)$ or $\left(n_{1}^{i},0\right)$, and wide $\left(n_{2}^{i}{,m}_{2}^{i}\right)$ or $\left(n_{2}^{i},0\right)$ SWCNTs, and is equal to 1, 2, 3, 4, 5, 8, 9, 10, 12 and 15. Each of these HJ sequences corresponds to a specific difference between diameters of the wide and narrow nanotubes, ∆*D* (Table 3 and Table 4). The selected range of ∆*D* of the HJ structures, described in the Table 2, Table 3 and Table 4, covers most cases of cone heterojunctions.

The tensile, (*EA*)*_HJ_*, bending, (*EI*)*_HJ_*, and torsional, (*GJ*)*_HJ_*, rigidities were calculated with help of the Equations (9)–(11), respectively, for HJ structures in Table 2, Table 3 and Table 4. The lengths of the narrow and wide SWCNTs regions, *L*_1_ and *L*_2_ were considered both equal to 100 nm. The geometrical parameter a, defined on the Figure 3, was considered equal to *L*_3_ tan(*γ*), where *γ* = 12.7°, whatever the *D*_*n*1_ and *D*_*n*2_. The loading condition in which the force (moment) is applied on the wide nanotube was considered.

**Figure 10:**
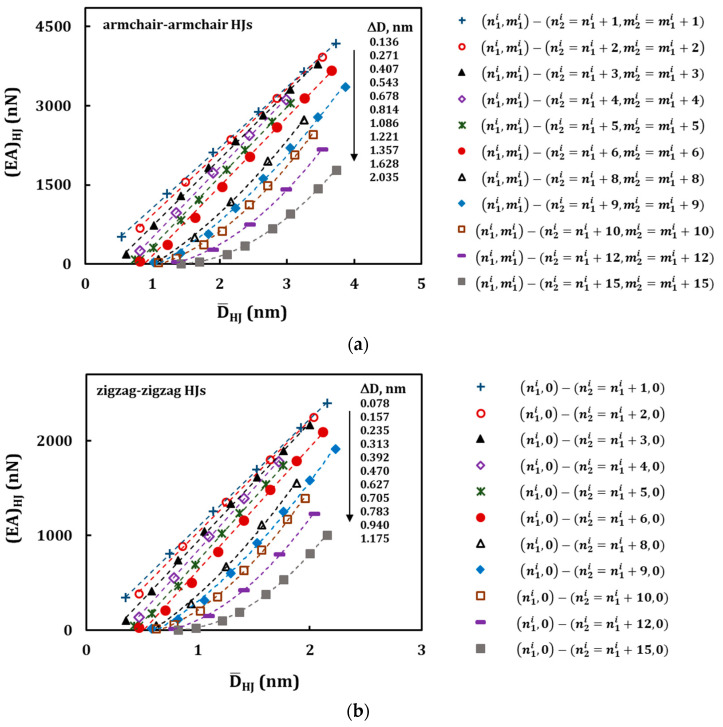
Evolutions of the tensile, (*EA*)*_HJ_*, rigidity with the average HJ diameter, ${\bar{D}}_{\mathrm{HJ}}$, for the HJs: (**a**) armchair–armchair (Table 3), and (**b**) zigzag–zigzag (Table 4). The force was applied to the wide SWCNT.

**Figure 11:**
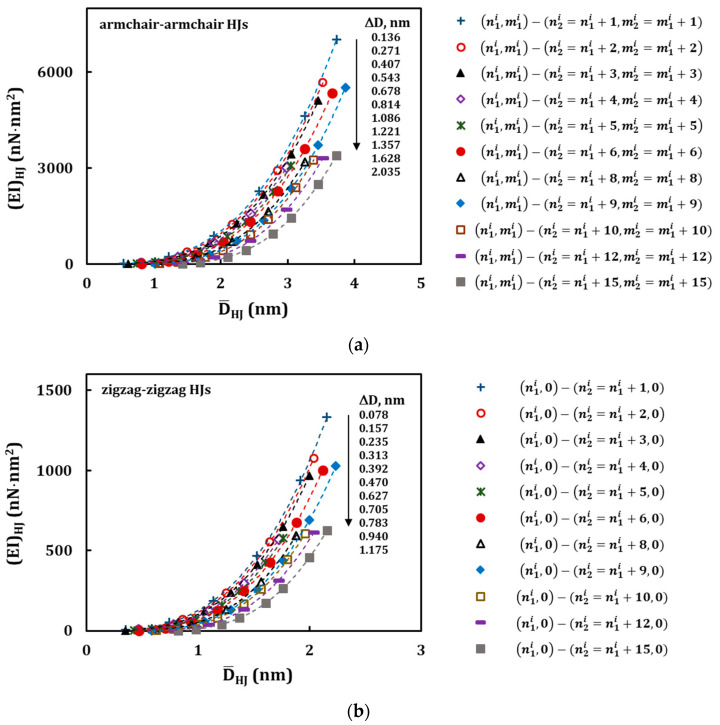
Evolutions of the bending, (*EI*)*_HJ_*, rigidity with the average HJ diameter, ${\bar{D}}_{\mathrm{HJ}}$, for the HJs: (**a**) armchair–armchair (Table 3) and (**b**) zigzag–zigzag (Table 4). The force was applied to the wide SWCNT.

**Figure 12:**
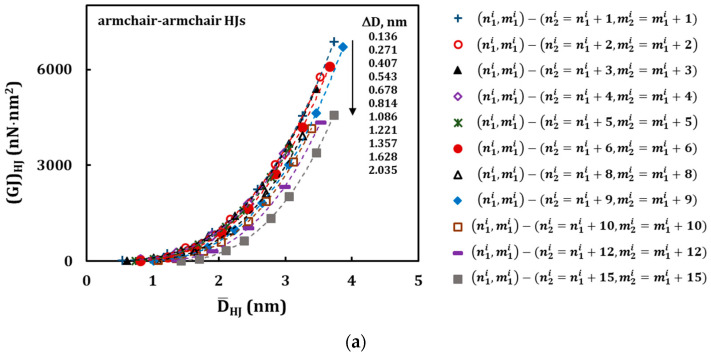

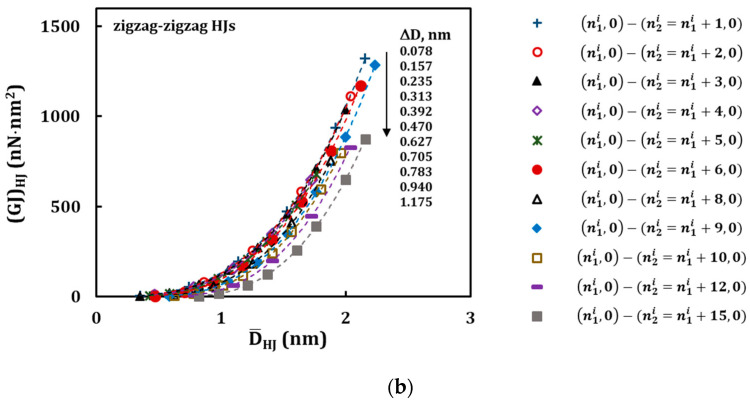
Evolutions of the torsional, (*GJ*)*_HJ_*, rigidity with the average HJ diameter, ${\bar{D}}_{\mathrm{HJ}}$, for the HJs: (**a**) armchair–armchair (Table 3) and (**b**) zigzag–zigzag (Table 4).

Similarly to Figure 8, the (*EA*)*_HJ_*, (*EI*)*_HJ_*, and (*GJ*)*_HJ_* values obtained analytically are plotted in Figure 10, Figure 11 and Figure 12, respectively, against the average HJ diameter ${\bar{D}}_{\mathrm{HJ}}$ for armchair–armchair (Figure 1a,Figure 11a and Figure 12a) and zigzag–zigzag (Figure 10b, 11b and 12b) HJs. The evolutions of the (*EA*)*_HJ_* rigidity follow a quasi-linear trend for HJs with ∆*D* up to 0.814 nm, for armchair–armchair, and 0.470 nm, for zigzag–zigzag structures, which correspond to the difference between the chiral indices of the wide and narrow nanotubes, *C* = 6 (Figure 10).

From the values of ∆*D* ≥ 1.086 nm, for armchair–armchair, and ∆*D* ≥ 0.627 nm, for zigzag–zigzag HJs, or *C* = 8, the (*EA*)*_HJ_* evolutions follow a second degree polynomial trend. These latter values of ∆*D* or *C* define the geometrical characteristics of HJ structures, from which the redundant bending deformation is observed, when analysing the results of numerical tensile test. That is, for HJ structures with ∆*D* ≤ 0.814 nm (armchair–armchair) and ∆*D* ≤ 0.470 nm (zigzag–zigzag), or when the difference between the chiral indices of the wide and narrow nanotubes is 1 ≤ *C* ≤ 6, the bending deformation that occurs during tensile test can be neglected: the evolution of the tensile rigidity with the average diameter follows an almost linear trend as in the case of the individual SWCNTs [45]. In Figure 11 and Figure 12, the evolutions of the bending, (*EI*)*_HJ_*, and torsional, (*GJ*)*_HJ_*, rigidities with the average HJ diameter, ${\bar{D}}_{\mathrm{HJ}}$, are close a third degree polynomial trend.

The equations fitted to the results of Figure 10, Figure 11 and Figure 12, which consist of first and second degree polynomials for tensile, (*EA*)*_HJ_* rigidity (Figure 10), and a third degree polynomial for bending (Figure 11), (*EI*)*_HJ_*, and torsional (Figure 12), (*GJ*)*_HJ_*, rigidities, are outlined in Appendix A (see Table A2 for armchair–armchair HJs, and Table A3 for zigzag–zigzag HJs). In these tables, each set of fitting equations for the (*EA*)*_HJ_*, (*EI*)*_HJ_* and (*GJ*)*_HJ_* rigidities concerns a specific difference between the diameters of the wide and narrow nanotubes, ∆*D*. In case of the tensile rigidity (*EA*)*_HJ_*, the fitting equations comprise second degree polynomials, for the highest ∆*D* values, when the redundant bending deformation in tension reaches importance. The fitting equations in Table A2 and Table A3 are alternatives to Equations (9)–(11), when used to assess the (*EA*)*_HJ_*, (*EI*)*_HJ_* and (*GJ*)*_HJ_* rigidities of HJs without resorting to numerical simulation, as long as the average HJ diameter, ${\bar{D}}_{\mathrm{HJ}}$, is known. The equations in Table A2 and Table A3 include polynomials of first and, in certain cases, second degree for the tensile rigidity, (*EA*)*_HJ_*, as the latter better describes the behaviour of this rigidity when redundant bending deformation occurs during the tensile tests.

It is worth noting that the almost linear dependence for the evolutions of (*EA*)*_HJ_* rigidity can be understood based on the linear relationship between heterojunction cross-section area, *A_HJ_*, and the HJ average diameter, ${\bar{D}}_{\mathrm{HJ}}$, considering the HJ structure as an equivalent SWCNT with diameter given by ${\bar{D}}_{\mathrm{HJ}}=\frac{1}{2}(D_{n1}+D_{n2})$:

(15) $$A_{\mathrm{HJ}}=\frac{\pi }{4}[{\left({\bar{D}}_{\mathrm{HJ}}+t_{n}\right)}^{2}-{\left({\bar{D}}_{\mathrm{HJ}}-t_{n}\right)}^{2}]=\pi {\bar{D}}_{\mathrm{HJ}}t_{n}$$

where *t_n_* = 0.34 nm is the wall thickness of the nanotube.

In turn, the third degree polynomial trend for the evolutions of (*EI*)*_HJ_* and (*GJ*)*_HJ_* with ${\bar{D}}_{\mathrm{HJ}}$ can be understood from the quasi-cubic relationship between the moments of inertia (in bending and torsion) and the average HJ diameter, ${\bar{D}}_{\mathrm{HJ}}$:

(16) $$I_{\mathrm{HJ}}=\frac{\pi }{64}[{\left({\bar{D}}_{\mathrm{HJ}}+t_{n}\right)}^{4}-{\left({\bar{D}}_{\mathrm{HJ}}-t_{n}\right)}^{4}]=\frac{\pi {\bar{D}}_{\mathrm{HJ}}^{3}t_{n}}{8}[1+{\left(\frac{t_{n}}{{\bar{D}}_{\mathrm{HJ}}}\right)}^{2}]$$

(17) $$J_{\mathrm{HJ}}=\frac{\pi }{32}[{\left({\bar{D}}_{\mathrm{HJ}}+t_{n}\right)}^{4}-{\left({\bar{D}}_{\mathrm{HJ}}-t_{n}\right)}^{4}]=\frac{\pi {\bar{D}}_{\mathrm{HJ}}^{3}t_{n}}{4}[1+{\left(\frac{t_{n}}{{\bar{D}}_{\mathrm{HJ}}}\right)}^{2}]$$

### 3.2. Comparison with Literature Results

The literature results for Young’s and shear moduli are summarised in Table 5. Most studies on the elastic properties of HJs reported in the literature are dealing with the evaluation of Young’s modulus of cone-heterojunctions [27,30,34,35,36,38]. The discrepancies observed in the values of the Young’s modulus of HJs (Table 5) can be related to different modelling and calculation approaches used. For example, when assessing the Young’s modulus of HJs from the results of the numerical tensile test, the various authors did not use the same approach in calculating the cross-sectional area of the heterojunction structure [30,35,38]. In fact, the definition of the elastic moduli of a HJ requires the concept of an equivalent nanotube, whose diameter value is intermediate between those of the two nanotubes that make up the HJ. The definition of the equivalent nanotube is not consensual among the various authors. In this context, it seems more appropriate not to present results of the elastic moduli (Young’s and shear modulus) and perform a comparison with literature results in terms of HJ rigidities.

The comparison study was limited by the works which comprise a definition of the equivalent cross-section area of the HJ structure. Thus, works where the Young’s modulus of HJs was obtained from the results of tensile test *E_HJ_* = (*EA*)*_HJ_*/*A_HJ_*, allow for making some comparisons [35,38]. Yengejeh et al. [38], using the assumption of HJ structure consisting of a sequence of springs, determined the equivalent cross-sectional area of the HJ, *A_HJ_*, as follows:

(18) $$A_{\mathrm{HJ}}=\frac{{2A}_{1}A_{2}}{\left(A_{1}+A_{2}\right)}$$

where, *A_1_* and *A_2_* are the cross-sectional areas of SWCNTs that constitute the heterojunction. Ghavamian and Ochsner [35] assumed the heterojunction as homogeneous SWCNTs having a cross-section area, $A_{\mathrm{HJ}}$, estimated by a weighted average, as follows:

(19) $$A_{\mathrm{HJ}}=\frac{L_{1}A_{1}{+L}_{3}A_{3}{+L}_{2}A_{2}}{L_{\mathrm{HJ}}}$$

where, *A_1_*, *A_2_* and *A_3_* are the cross-sectional areas of constituent SWCNTs and the connecting region, respectively; *L*_1_, *L*_2_ are the lengths of the narrow and wide SWCNTs constituting HJ, respectively, and *L*_3_ is the length of the connecting region; *L_HJ_* = *A*_1_ + *A*_2_ + *A*_3_ is the overall length of HJs. The cross-sectional area of connecting region, *A*_3_, is given by:

(20) $$A_{3}=\frac{\left(A_{1}{+A}_{2}\right)}{2}$$

The tensile rigidity of HJs is determined by:

(21) $${\left(\mathrm{EA}\right)}_{\mathrm{HJ}}{=E}_{\mathrm{HJ}}A_{\mathrm{HJ}}$$

where E_HJ_ is the HJ Young’s modulus, taken from the literature, *A_HJ_* is the HJ cross-sectional area determined by Equations (18) or (19), depending on the work from which it was taken.

Table 6 compares the values of (*EA*)*_HJ_* evaluated from the Young’s modulus results of the works [35,38] using Equation (21), considering the respective expressions (Equation (18) or Equation (19)) for calculating the HJ cross-section area, *A_HJ_*, with current tensile rigidity results obtained analytically using Equations (9) and (12)–(14), which allows easy comparison with results from those works. The average difference between current (*EA*)*_HJ_* values and those obtained in the works by Yengejeh et al. [38], and Ghavamian and Ochsner [35] is 1.62% and 3.03%, respectively. This reinforces the reliability of the analytical approach involving Equations (9) and (12)–(14), recommended in this study for determining the tensile rigidity of cone-heterojunctions, without resorting to numerical simulation, as long as the geometrical parameters of the HJs are known.

**Figure 13:**
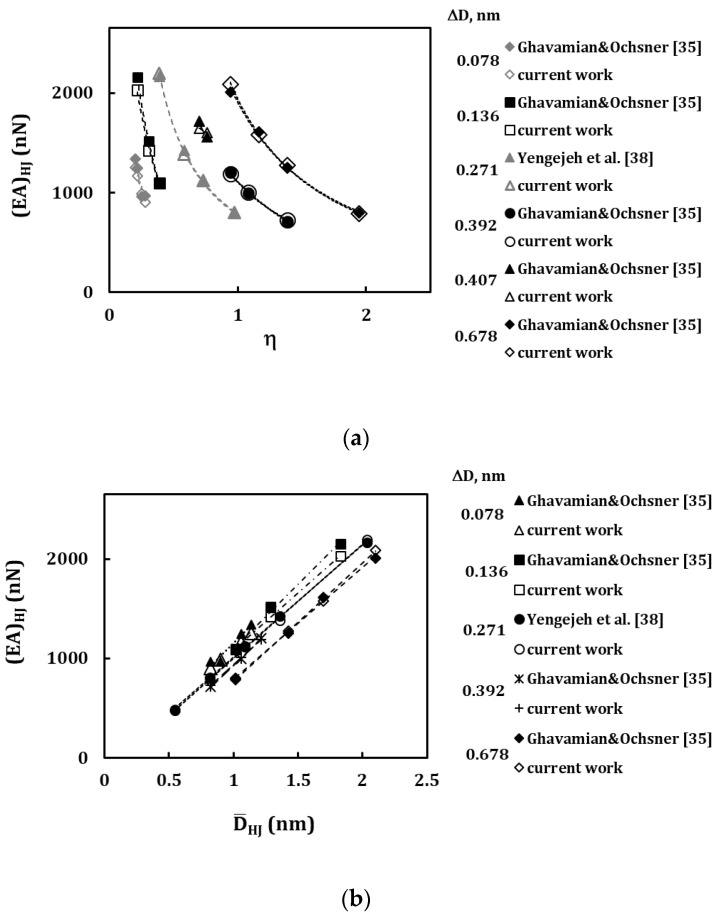
Comparison of the evolutions of tensile rigidity, (*EA*)*_HJ_*, (**a**) with the HJ aspect ratio, *η*, and (**b**) with the average HJ diameter, D_HJ_. Each sequence (i.e., line) considers HJs with a given difference between the diameters of the wide and narrow nanotubes, ∆*D*.

To complement and make it easy to understand the comparative analysis on the HJ tensile rigidity, some results in Table 6 were plotted as a function of the heterojunction aspect ratio, *η* (Figure 13a), and as a function of the average HJ diameter, D_HJ_ (Figure 13b). In Figure 13 the results are grouped (see lines in the figures) for HJ sequences that have the same difference between the values of the diameters of the wide and narrow nanotubes. i.e., ∆*D* = 0.136, 0.271, 0.407, 0.678 nm for armchair–armchair HJs, ∆*D* = 0.078, 0.392 nm for zigzag–zigzag HJs. A good correspondence with the literature results is observed. The evolutions of the tensile (*EA*)*_HJ_* rigidity with the HJ aspect ratio, η, obtained in the present study and those assessed by Yengejeh et al. [38] and Ghavamian and Ochsner [35] follow similar trends. The same is true for the evolutions of the tensile rigidity, (*EA*)*_HJ_*, with the average HJ diameter, $D_{\mathrm{HJ}}$.

To our knowledge, only a few papers have been devoted to the evaluation of the shear modulus of HJs [27,35,36]. In case of the shear modulus, the results available in the literature are less than for the Young’s modulus and have little consistency (Table 5). Results of two works, by Ghavamian and Ochsner [35] and Hemmatian et al. [36], were chosen for comparison purposes. In both studies [35,36], the NCM modelling approach was employed, and the HJ shear modulus was calculated from the results of torsional test of the HJ structure, seen as an equivalent SWCNT with the average diameter ${\bar{D}}_{\mathrm{HJ}}$ (given by Equation (2)).

In order to perform a comparison with literature results in terms of HJ rigidity, the torsional rigidity of the HJs was determined from the values of the shear modulus reported by Ghavamian and Ochsner [35], and Hemmatian et al. [36], as follows:

(22) $${\left(\mathrm{GJ}\right)}_{\mathrm{HJ}}=G_{\mathrm{HJ}}J_{\mathrm{HJ}}$$

where (*GJ*)*_HJ_* is the HJ shear modulus, and *J_HJ_* is calculated from Equation (17) considering that ${\bar{D}}_{\mathrm{HJ}}$ is the average diameter of the nanotubes constituting the HJ, and $t_{n}=0.34\mathrm{nm}$.

**Figure 14:**
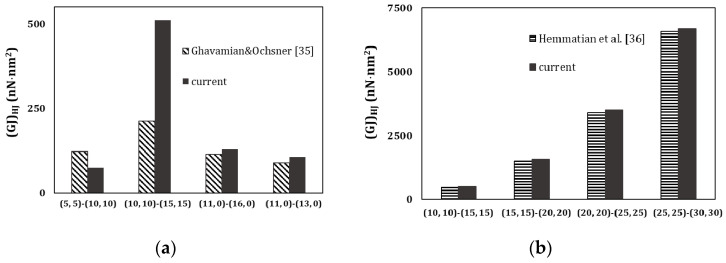
Comparison between the current results of torsional rigidity of HJs, (*GJ*)*_HJ_*, with those calculated from the works by (**a**) Ghavamian and Ochsner [35] and (**b**) Hemmatian et al. [36]. The (*GJ*)*_HJ_* values for [35,36] were calculated using Equation (22).

**Figure 15:**
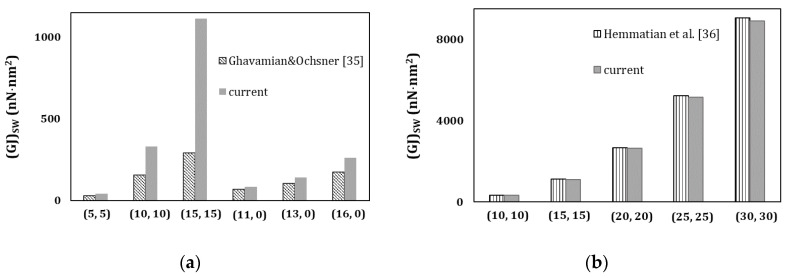
Comparison between the current results of torsional rigidity, (*GJ*)_*SW*_, of individual SWCNTs, with those calculated from the works by (**a**) Ghavamian and Ochsner [35] and (**b**) Hemmatian et al. [36].

Figure 14 compares the current (*GJ*)*_HJ_* results, obtained from the FE analysis, with the results calculated from the works of Ghavamian and Ochsner [35] (Figure 14a) and Hemmatian et al. [36] (Figure 14b). A certain discrepancy is observed between the current values of (*GJ*)*_HJ_* and those calculated from the work of Ghavamian and Ochsner [35], although a good correspondence is found when the current torsional rigidity results are compared with those calculated from Hemmatian et al. [36]. The average difference between the (*GJ*)*_HJ_* values taken from FE analysis in the present study and those obtained from the results of Hemmatian et al. [36] is 3.95%. It should be noted that when discrepancy occurs between the current results of torsional rigidity of HJs and those obtained by Ghavamian and Ochsner [35], this also happens for the torsional rigidities of individual SWCNTs; when the discrepancy is not significant for the HJs, it is also not significant for individual SWCNTs. This can be concluded from Figure 15a,b, which shows the torsional rigidities, (*GJ*)*_HJ_*, of the individual SWCNTs constituting the HJs of Figure 14a,b, respectively.

## 4. Conclusions

A three-dimensional finite element model of cone-heterojunctions was used in order to carry out a systematic evaluation of the tensile, bending and torsional rigidities of armchair–armchair and zigzag–zigzag HJs.

The main conclusions of this comprehensive study are as follows:

- For heterojunction with the same difference between the diameters of the wide and narrow nanotubes, ∆*D*, the tensile rigidity decreases with the increase of the HJ aspect ratio, following an exponential trend. The same type of trend occurs for the evolutions of the bending and torsional rigidities with the HJ aspect ratio.
- The tensile, bending and torsional rigidities increase with the increase of the average HJ diameter. The evolutions of the tensile rigidity follow a quasi-linear trend for heterojunctions with ∆*D* = 0.678 nm (armchair–armchair) and ∆*D* = 0.392 nm (zigzag–zigzag); but for greater differences between the diameters of the wide and narrow nanotubes, the tensile rigidity evolution are close to a second degree polynomial trend; this different behaviour is most likely linked to the occurrence of redundant bending deformation during the tensile test. The evolutions of the bending and torsional rigidities with the average HJ diameter are close a third degree polynomial trend.
- Equations (9)–(11) (together with Equations (12)–(14)) offer a robust method for easily assessing the tensile, bending and torsional rigidities of HJs in a wide range of their geometrical parameters, were recommended. The accuracy of these analytical solutions was successfully tested on current results as well those available in the literature.

It should be noted that taking into account promising potential applications of the SWCNT HJs and the impossibility of producing the ideal (without defects) HJ structure, a comprehensive study concerning the influence of structural imperfections on the elastic properties of the SWCNT HJs is under consideration for future studies.

## Figures and Tables

**Table 1:** Material and geometrical properties of the beam element used as input parameters for FE simulations of SWCNT HJs.

Parameter	Value	Formulation
Bond stretching force constant, *k_r_* [43]	6.52 × 10^−7^ N·nm^−1^	–
Bond bending force constant, *k_θ_* [43]	8.76 × 10^−10^ N·nm·rad^−2^	–
Torsional resistance force constant, *k_τ_* [43,44]	2.78 × 10^−10^ N·nm·rad^−2^	–
C–C bond/beam length (*l* = *a*_c-c_)	0.1421 nm	–
Diameter (*d*)	0.147 nm	$d=4\sqrt{k_{\theta }/k_{r}}$
Cross section area, *A_b_*	0.01688 nm^2^	$A_{b}{=\pi d}^{2}/4$
Moment of inertia, *I_b_*	2.269 × 10^−5^ nm^4^	$I_{b}{=\pi d}^{4}/64$
Polar moment of inertia, *J_b_*	4.53 7 × 10^−5^ nm^4^	$J_{b}{=\pi d}^{4}/32$
Young’s modulus, *E_b_*	5488 GPa	$E_{b}{=k}_{r}^{2}l/{4\pi k}_{\theta }$
Shear modulus, *G_b_*	870.7 GPa	$G_{b}=k_{r}^{2}k_{\tau }l/{8\pi k}_{\theta }^{2}$
Rigidity, *E_b_A_b_*	92.65 nN	$E_{b}A_{b}{=k}_{r}l$
Rigidity, *E_b_I_b_*	0.1245 nN·nm^2^	$E_{b}I_{b}{=k}_{\theta }l$
Rigidity, G_b_J_b_	0.0395 nN·nm^2^	$G_{b}J_{b}{=k}_{\tau }l$

**Table 2:** Geometrical characteristics of the HJs under study.

HJ	(n_1_, m_1_)–(n_2_, m_2_)	Δ*D*, nm	${\bar{D}}_{HJ},\mathrm{nm}$	*η*	*L*_1_, nm	*L*_2_, nm	*L*_3_, nm
armchair–armchair	(5, 5)–(10, 10)	0.678	1.018	1.940	100.01	99.95	1.97
	(5, 5)–(15, 15)	1.357	1.357	2.912	100.01	100.02	3.95
	(5, 5)–(20, 20)	2.035	1.696	3.496	100.01	100.04	5.93
	(10, 10)–(15, 15)	0.678	1.696	1.166	100.06	100.00	1.98
	(10, 10)–(20, 20)	1.357	2.035	1.943	100.05	100.07	3.96
	(10, 10)–(25, 25)	2.035	2.375	2.501	100.04	100.06	5.94
	(15, 15)–(20, 20)	0.678	2.375	0.833	100.00	100.01	1.98
	(15, 15)–(25, 25)	1.357	2.714	1.458	100.02	99.98	3.96
	(15, 15)–(30, 30)	2.035	3.053	1.946	100.02	99.97	5.94
	(20, 20)–(25, 25)	0.678	3.053	0.649	100.00	101.99	1.98
	(20, 20)–(30, 30)	1.357	3.392	1.167	100.02	99.99	3.96
	(20, 20)–(35, 35)	2.035	3.732	1.592	100.04	99.97	5.94
zigzag–zigzag	(5, 0)–(10, 0)	0.392	0.588	1.950	99.92	99.96	1.15
	(5, 0)–(15, 15)	0.783	0.783	2.918	100.11	100.13	2.29
	(5, 0)–(20, 20)	1.175	0.979	3.500	100.12	100.10	3.43
	(10, 0)–(15, 0)	0.392	0.979	1.177	100.14	100.12	1.15
	(10, 0)–(20, 0)	0.783	1.175	1.952	99.97	100.10	2.29
	(10, 0)–(25, 0)	1.175	1.371	2.502	99.96	100.10	3.43
	(15, 0)–(20, 0)	0.392	1.371	0.843	100.03	100.00	1.16
	(15, 0)–(25, 0)	0.783	1.567	1.466	100.02	99.97	2.30
	(15, 0)–(30, 0)	1.175	1.763	1.952	100.01	99.94	3.44
	(20, 0)–(25, 0)	0.392	1.763	0.647	99.94	104.61	1.14
	(20, 0)–(30, 0)	0.783	1.959	1.169	99.92	102.34	2.29
	(20, 0)–(35, 0)	1.175	2.154	1.597	99.92	100.05	3.44

**Table 3:** Geometrical characteristics of armchair–armchair HJs used to test the analytical model.

(n_1_, m_1_)–(n_2_, m_2_)	*C* *	Δ*D*, nm	${\bar{D}}_{HJ},\mathrm{nm}$	(n_1_, m_1_)–(n_2_, m_2_)	*C* *	Δ*D*, nm	${\bar{D}}_{HJ},\mathrm{nm}$
(4, 4)–(5, 5)	1	0.136	0.543	(5, 5)–(7, 7)	2	0.271	0.814
(9, 9)–(10, 10)			1.221				
(14, 14)–(15, 15)			1.900	(10, 10)–(12, 12)			1.493
(19, 19)–(20, 20)			2.578	(15, 15)–(17, 17)			2.171
(24, 24)–(25, 25)			3.257	(20, 20)–(22, 22)			2.850
(27, 27)–(28, 28)			3.732	(25, 25)–(27, 27)			3.528
(3, 3)–(6, 6)	3	0.407	0.611	(4, 4)–(8, 8)	4	0.543	0.814
(6, 6)–(9, 9)			1.018				
(9, 9)–(12, 12)			1.425	(8. 8)–(12, 12)			1.357
(12, 12)–(15, 15)			1.832	(12, 12)–(16, 16)			1.900
(15, 15)–(18, 18)			2.239				
(18, 18)–(21, 21)			2.646	(16, 16)–(20, 20)			2.443
(21, 21)–(24, 24)			3.053	(20, 20)–(24, 24)			2.985
(24, 24)–(27, 27)			3.460				
(3, 3)–(8, 8)	5	0.678	0.746	(3, 3)–(9, 9)	6	0.814	0.814
				(6, 6)–(12, 12)			1.221
(8, 8)–(13, 13)			1.425	(9, 9)–(15, 15)			1.628
				(12, 12)–(18, 18)			2.035
(13, 13)–(18, 18)			2.103	(15, 15)–(21, 21)			2.443
				(18, 18)–(24, 24)			2.850
(18, 18)–(23, 23)			2.782	(21, 21)–(27, 27)			3.257
				(24, 24)–(30, 30)			3.664
(4, 4)–(12, 12)	8	1.086	1.086	(3, 3)–(12, 12)	9	1.221	1.018
				(6, 6)–(15, 15)			1.425
(8, 8)–(16, 16)			1.628	(9, 9)–(18, 18)			1.832
(12, 12)–(20, 20)			2.171	(12, 12)–(21, 21)			2.239
				(15, 15)–(24, 24)			2.646
(16, 16)–(24, 24)			2.714	(18, 18)–(27, 27)			3.053
(20, 20)–(28, 28)			3.257	(21, 21)–(30, 30)			3.460
				(24, 24)–(33, 33)			3.867
(3, 3)–(13, 13)	10	1.357	1.086	(4, 4)–(16, 16)	12	1.628	1.357
				(8, 8)–(20, 20)			1.900
(8, 8)–(18, 18)			1.764	(12, 12)–(24, 24)			2.443
(13, 13)–(23, 23)			2.443	(16, 16)–(28, 28)			2.985
(18, 18)–(28, 28)			3.121	(20, 20)–(32, 32)			3.528
(3, 3)–(18, 18)	15	2.035	1.425	–	–	–	–
(8, 8)–(23, 23)			2.103	–	–	–	–
(13, 13)–(28, 28)			2.782	–	–	–	–
(18, 18)–(33, 33)			3.460	–	–	–	–

* The constant C is the difference between the chiral indices of the wide $\left(n_{1}^{i}{,m}_{1}^{i}\right)$, and narrow $\left(n_{2}^{i}{,m}_{2}^{i}\right)$ SWCNTs, constituting the heterojunction: $\left(n_{1}^{i},m_{1}^{i}\right)–(n_{2}^{i}=n_{1}^{i}+C,m_{2}^{i}=m_{1}^{i}+C)$.

**Table 4:** Geometrical characteristics of zigzag–zigzag HJs used to test the analytical model.

(n_1_, 0)–(n_2_, 0)	*C* *	Δ*D*, nm	${\bar{D}}_{HJ},\mathrm{nm}$	(n_1_, 0)–(n_2_, 0)	*C* *	Δ*D*, nm	${\bar{D}}_{HJ},\mathrm{nm}$
(4, 0)–(5, 0)	1	0.078	0.353	(5, 0)–(7, 0)	2	0.157	0.470
(9, 0)–(10, 0)			0.744				
(14, 0)–(15, 0)			1.136	(10, 0)–(12, 0)			0.862
(19, 0)–(20, 0)			1.528	(15, 0)–(17, 0)			1.254
(24, 0)–(25, 0)			1.919	(20, 0)–(22, 0)			1.645
(27, 0)–(28, 0)			2.154	(25, 0)–(27, 0)			2.037
(3, 0)–(6, 0)	3	0.235	0.353	(4, 0)–(8, 0)	4	0.133	0.470
(6, 0)–(9, 0)			0.588				
(9, 0)–(12, 0)			0.823	(8. 0)–(12, 0)			0.783
(12, 0)–(15, 0)			1.058	(12, 0)–(16, 0)			1.097
(15, 0)–(18, 0)			1.293				
(18, 0)–(21, 0)			1.528	(16, 0)–(20, 0)			1.410
(21, 0)–(24, 0)			1.763	(20, 0)–(24, 0)			1.724
(24, 0)–(27, 0)			1.998				
(3, 0)–(8, 0)	5	0.392	0.431	(3, 0)–(9, 0)	6	0.470	0.470
				(6, 0)–(12, 0)			0.705
(8, 0)–(13, 0)			0.823	(9, 0)–(15, 0)			0.940
				(12, 0)–(18, 0)			1.175
(13, 0)–(18, 0)			1.214	(15, 0)–(21, 0)			1.410
				(18, 0)–(24, 0)			1.645
(18, 0)–(23, 0)			1.606	(21, 0)–(27, 0)			1.880
				(24, 0)–(30, 0)			2.115
(4, 0)–(12, 12)	8	0.627	0.627	(3, 0)–(12, 0)	9	0.705	0.588
				(6, 0)–(15, 0)			0.823
(8, 0)–(16, 16)			0.940	(9, 0)–(18, 0)			1.058
(12, 0)–(20, 20)			1.254	(12, 0)–(21, 0)			1.293
				(15, 0)–(24, 0)			1.528
(16, 0)–(24, 24)			1.567	(18, 0)–(27, 0)			1.763
(20, 0)–(28, 28)			1.880	(21, 0)–(30, 0)			1.998
				(24, 0)–(33, 0)			2.233
(3, 0)–(13, 0)	10	0.783	0.627	(4, 0)–(16, 0)	12	0.940	0.783
				(8, 0)–(20, 0)			1.097
(8, 0)–(18, 0)			1.018	(12, 0)–(24, 0)			1.410
(13, 0)–(23, 0)			1.410	(16, 0)–(28, 0)			1.724
(18, 0)–(28, 0)			1.802	(20, 0)–(32, 0)			2.037
(3, 0)–(18, 0)	15	1.175	0.823	-			
(8, 0)–(23, 0)			1.214				
(13, 0)–(28, 0)			1.606				
(18, 0)–(33, 0)			1.998				

* The constant *C* is the difference between the chiral indices of the wide $\left(n_{1}^{i},0\right)$, and narrow $\left(n_{2}^{i},0\right)$ SWCNTs, constituting the heterojunction: $\left(n_{1}^{i},0\right)–(n_{2}^{i}=n_{1}^{i}+C,0)$.

**Table 5:** Elastic moduli of cone-heterojunctions available in the literature.

Reference	Method	Type of HJs	Young’s Modulus, *E_HJ_*, TPa	Shear Modulus, *G_HJ_*, TPa
Qin et al. [30]	Molecular dynamics (MD): Tersoff–Brenner potential	armchair–armchair sequence with narrow SWCNT (5, 5)	0.775 *	–
		zigzag–zigzag sequence with narrow SWCNT (9, 0)	0.795 *	–
Scarpa et al. [34]	Nanoscale continuum modelling (NCM): linear beams	armchair–armchair (5, 5)–(10, 10)	1.010	–
		zigzag–zigzag (9, 9)–(14, 14)	0.945	–
Hemmatian et al. [36]		armchair–armchair (5, 5)–(10, 10), (10, 10)–(15, 15)…(25, 25)–(30, 30)	1.109 *	0.344 *
Yengejeh et al. [38]		armchair–armchair (5, 5)–(10, 10) and a sequence with Δ*D* = 0.271 nm	0.947 *	–
		zigzag–zigzag (11, 0)–(12, 0),(9, 0)–(12, 0), (12, 0)–(16, 0)	0.982 *	–
Ghavamian and Ochsner [35], Ghavamian et al. [27]		armchair–armchair composed by SWCNTs in a range of (7, 7) to (18, 18)	0.927 *	0.180 *
		zigzag–zigzag composed by SWCNTs in a range of (6, 0) to (18, 0)	0.939 *	0.270 *

* Average value.

**Table 6:** Comparison of current (*EA*)*_HJ_* results with those from the literature.

Reference	(n_1_, m_1_)–(n_2_, m_2_) (n_1_, 0)–(n_2_, 0)	*η*	Δ*D*, nm	${\bar{D}}_{HJ},\mathrm{nm}$	*E_HJ_*, Tpa Reference	(*EA*)*_HJ_*, nN		Difference, %
						Reference + Equation (21)	Current	
Yengejeh et al. [38]	(3, 3)–(5, 5)	1.458	0.271	0.543	0.889	483.20	474.16	1.87
	(5, 5)–(7, 7)	0.972		0.814	0.953	805.76	796.82	1.11
	(7, 7)–(9, 9)	0.729		1.086	0.981	1119.73	1118.61	0.10
	(9, 9)–(11, 11)	1.944		1.357	0.992	1423.45	1381.59	2.94
	(14, 14)–(16, 16)	0.583		2.035	1.001	2166.63	2196.91	1.40
	(5, 5)–(10, 10)	0.389	0.678	1.018	0.865	835.83	786.07	5.95
	(9, 0)–(12, 0)	0.833	0.235	0.823	0.960	826.30	823.72	0.31
	(12, 0)–(16, 0)	0.833	0.313	1.097	0.962	1104.03	1099.54	0.41
	(11, 0)–(12, 0)	0.254	0.078	0.901	1.023	982.62	987.69	0.52
Ghavamian and Ochsner [35]	(8, 8)–(15, 15)	1.775	0.950	1.560	0.765	1275.13	1274.97	0.01
	(5, 5)–(10, 10)	1.944	0.678	1.018	0.735	798.99	786.07	1.62
	(8, 8)–(13, 13)	1.388		1.425	0.825	1255.56	1273.08	1.40
	(10, 10)–(15, 15)	1.166		1.696	0.889	1610.67	1577.30	2.07
	(13, 13)–(18, 18)	0.941		2.103	0.895	2010.71	2085.06	3.70
	(10, 10)–(13, 13)	0.761	0.407	1.560	0.935	1558.49	1600.93	2.72
	(11, 11)–(14, 14)	0.700		1.696	0.945	1712.13	1647.32	3.79
	(7, 7)–(8, 8)	0.389	0.136	1.018	1.001	1088.15	1096.29	0.75
	(9, 9)–(10, 10)	0.307		1.289	1.101	1516.02	1420.02	6.33
	(13, 13)–(14, 14)	0.216		1.832	1.101	2154.35	2032.28	5.67
	(9, 0)–(19, 0)	2.083	0.783	1.097	0.678	794.31	823.08	3.62
	(13, 0)–(8, 0)	1.388	0.392	0.823	0.810	711.72	726.71	2.11
	(11, 0)–(16, 0)	1.080		1.058	0.880	994.15	1004.90	1.08
	(13, 0)–(18, 0)	0.941		1.214	0.925	1199.80	1190.67	0.76
	(11, 0)–(13, 0)	0.486	0.157	0.940	0.978	982.10	980.93	0.12
	(10, 0)–(11, 0)	0.278	0.078	0.823	1.101	967.41	902.63	6.70
	(11, 0)–(12, 0)	0.254		0.901	1.001	963.31	987.69	2.53
	(13, 0)–(14, 0)	0.216		1.058	1.101	1243.81	1165.03	6.33
	(14, 0)–(15, 0)	0.201		1.136	1.101	1335.95	1253.40	6.18

## Appendix A

**Table A1:** Comparison of the tensile, (*EA*)*_HJ_*, bending, (*EI*)*_HJ_*, and torsional, (*GJ*)*_HJ_* rigidities of HJs obtained from the FE analysis (and using Equations (6)–(8)) and those analytically calculated from Equations (9)–(11).

Force/Moment Application	(n_1_, m_1_)–(n_2_, m_2_) (n_1_, 0)–(n_2_, 0)	*η*	${\bar{D}}_{\mathrm{HJ}},\mathrm{nm}$	Δ*D*, nm	(*EA*)*_HJ_* nN Equation (6)	(*EA*)*_HJ_* nN Equation (9)	Difference %	(*EI*)*_HJ_* nN·nm^2^ Equation (7)	(*EI*)*_HJ_* nN·nm^2^ Equation (10)	Difference %	(*GJ*)*_HJ_* nN·nm^2^ Equation (8)	(*GJ*)*_HJ_* nN·nm^2^ Equation (11)	Difference %
force (moment) applied on wide SWCNT	(5, 5)–(10, 10)	1.940	1.018	0.678	308.04	308.36	0.10	50.24	51.25	2.01	74.20	72.04	2.91
	(5, 5)–(15, 15)	2.912	1.357	1.357	101.61	101.65	0.04	51.07	53.44	4.65	81.17	78.81	2.91
	(5, 5)–(20, 20)	3.496	1.696	2.035	48.11	48.10	0.02	51.41	55.19	7.34	83.65	81.21	2.91
	(10, 10)–(15, 15)	1.166	1.696	0.678	1194.04	1206.19	1.02	384.92	396.14	2.92	510.75	511.75	0.19
	(10, 10)–(20, 20)	1.943	2.035	1.357	609.46	618.36	1.46	394.96	417.75	5.77	591.45	595.34	0.66
	(10, 10)–(25, 25)	2.501	2.375	2.035	306.53	335.28	9.38	411.65	433.78	5.38	628.44	634.98	1.04
	(15, 15)–(20, 20)	0.833	2.375	0.678	2125.05	2150.45	1.20	1270.42	1311.34	3.22	1567.89	1581.55	0.87
	(15, 15)–(25, 25)	1.458	2.714	1.357	1440.17	1468.64	1.98	1307.85	1388.88	6.20	1837.69	1864.30	1.45
	(15, 15)–(30, 30)	1.946	3.053	2.035	846.84	932.35	10.10	1376.04	1447.92	5.22	2013.14	2036.69	1.17
	(20, 20)–(25, 25)	0.649	3.053	0.678	2964.63	3044.91	2.71	2928.90	3073.60	4.94	3495.42	3531.26	1.03
	(20, 20)–(30, 30)	1.167	3.392	1.357	2377.33	2431.10	2.26	3188.52	3252.21	2.00	4107.84	4155.20	1.15
	(20, 20)–(35, 35)	1.592	3.732	2.035	1602.32	1757.05	9.66	3229.55	3398.61	5.23	4531.93	4583.19	1.13
	(5, 0)–(10, 0)	1.950	0.588	0.392	163.24	163.58	0.21	8.74	8.87	1.42	14.51	14.53	0.18
	(5, 0)–(15, 0)	2.918	0.783	0.783	52.86	53.17	0.58	8.78	9.12	3.94	15.71	15.88	1.10
	(5, 0)–(20, 0)	3.500	0.979	1.175	24.67	24.97	1.20	8.82	9.31	5.52	16.13	16.31	1.14
	(10, 0)–(15, 0)	1.177	0.979	0.392	675.81	682.64	1.01	72.34	73.37	1.42	98.57	98.85	0.28
	(10, 0)–(20, 0)	1.952	1.175	0.783	344.27	346.50	0.65	74.17	76.43	3.05	113.95	114.68	0.64
	(10, 0)–(25, 0)	1.444	2.375	1.175	171.53	187.89	9.54	76.40	78.34	2.54	120.67	121.78	0.92
	(15, 0)–(20, 0)	0.843	1.371	0.392	1219.63	1228.74	0.75	242.56	246.32	1.55	302.28	303.58	0.43
	(15, 0)–(25, 0)	1.466	1.567	0.783	827.05	833.80	0.82	249.33	257.66	3.34	354.59	356.61	0.57
	(15, 0)–(30, 0)	1.952	1.763	1.175	481.60	527.12	9.45	257.97	265.33	2.85	383.83	387.81	1.04
	(20, 0)–(25, 0)	0.647	1.763	0.392	1693.20	1754.72	3.63	565.87	582.17	2.88	675.82	681.70	0.87
	(20, 0)–(30, 0)	1.169	1.959	0.783	1383.69	1400.28	1.20	586.48	609.14	3.86	790.48	799.44	1.13
	(20, 0)–(35, 0)	1.597	2.154	1.175	927.74	999.98	7.79	596.09	627.10	5.20	858.58	873.75	1.77
force (moment) applied on narrow SWCNT	(5, 5)–(10, 10)	1.940	1.018	0.678	786.07	792.03	0.76	189.69	193.75	2.14	74.20	72.04	2.91
	(5, 5)–(15, 15)	2.912	1.357	1.357	820.72	835.91	1.85	289.11	297.89	3.04	81.17	78.81	2.91
	(5, 5)–(20, 20)	3.496	1.696	2.035	870.97	897.04	2.99	337.49	348.60	3.29	83.65	81.21	2.91
	(10, 10)–(15, 15)	1.166	1.696	0.678	1577.30	1592.94	0.99	909.80	938.67	3.17	510.75	511.75	0.19
	(10, 10)–(20, 20)	1.943	2.035	1.357	1565.47	1597.56	2.05	1499.19	1584.98	5.72	591.45	595.34	0.66
	(10, 10)–(25, 25)	2.501	2.375	2.035	1493.29	1641.25	9.91	2038.86	2115.75	3.77	629.64	634.98	0.85
	(15, 15)–(20, 20)	0.833	2.375	0.678	2381.49	2404.45	0.96	2376.88	2460.03	3.50	1567.89	1581.55	0.87
	(15, 15)–(25, 25)	1.458	2.714	1.357	2346.46	2400.72	2.31	3732.94	3995.31	7.03	1837.69	1864.30	1.45
	(15, 15)–(30, 30)	1.946	3.053	2.035	2226.74	2418.23	8.60	5255.92	5500.76	4.66	2016.10	2036.69	1.02
	(20, 20)–(25, 25)	0.649	3.053	0.678	3117.55	3212.24	3.04	4876.24	5056.65	3.70	3494.92	3531.26	1.04
	(20, 20)–(30, 30)	1.167	3.392	1.357	3118.00	3216.03	3.14	7365.60	7724.38	4.87	4111.25	4155.20	1.07
	(20, 20)–(35, 35)	1.592	3.732	2.035	2949.22	3223.37	9.30	10072.71	10594.98	5.19	4538.18	4583.19	0.99
	(5, 0)–(10, 0)	1.950	0.588	0.392	437.89	439.12	0.28	34.20	34.58	1.11	14.51	14.53	0.18
	(5, 0)–(15, 0)	2.918	0.783	0.783	458.46	463.58	1.12	50.49	51.70	2.40	15.71	15.88	1.10
	(5, 0)–(20, 0)	3.500	0.979	1.175	487.37	495.73	1.71	57.73	59.25	2.63	16.13	16.31	1.14
	(10, 0)–(15, 0)	1.177	0.979	0.392	899.77	906.30	0.73	172.67	175.45	1.61	98.57	98.85	0.28
	(10, 0)–(20, 0)	1.952	1.175	0.783	897.03	906.55	1.06	284.75	292.87	2.85	113.95	114.68	0.64
	(10, 0)–(25, 0)	1.444	2.375	1.175	847.80	929.46	9.63	368.71	384.04	4.16	120.39	121.78	1.15
	(15, 0)–(20, 0)	0.843	1.371	0.392	1370.79	1375.72	0.36	456.02	464.13	1.78	302.28	303.58	0.43
	(15, 0)–(25, 0)	1.466	1.567	0.783	1352.27	1367.86	1.15	717.56	743.36	3.60	354.59	356.61	0.57
	(15, 0)–(30, 0)	1.952	1.763	1.175	1258.81	1369.69	8.81	963.25	1007.87	4.63	383.33	387.81	1.17
	(20, 0)–(25, 0)	0.647	1.763	0.392	1895.37	1845.22	2.65	919.06	958.28	4.27	674.93	681.70	1.00
	(20, 0)–(30, 0)	1.169	1.959	0.783	1802.76	1834.45	1.76	1388.73	1459.07	5.07	791.16	799.44	1.05
	(20, 0)–(35, 0)	1.597	2.154	1.175	1659.32	1829.64	10.26	1857.78	1958.78	5.44	858.58	873.75	1.77

**Table A2:** Equations fitted to the results in Figure 10a, Figure 11a, and Figure 12a, for the cases of the tensile, (*EA*)*_HJ_*, bending, (*EI*)*_HJ_*, and torsional, (*GJ*)*_HJ_* rigidities of armchair–armchair HJs.

Δ*D*, nm	*C*	Rigid.	Fitting Equations
0.136	1	(*EA*)*_HJ_*	$1144.20{\bar{D}}_{\mathrm{HJ}}-79.79$
		(*EI*)*_HJ_*	$140.92{\bar{D}}_{\mathrm{HJ}}^{3}-21.543{\bar{D}}_{\mathrm{HJ}}^{2}-0.5184{\bar{D}}_{\mathrm{HJ}}+0.2604$
		(*GJ*)*_HJ_*	$132.22{\bar{D}}_{\mathrm{HJ}}^{3}+0.0569{\bar{D}}_{\mathrm{HJ}}^{2}-3.8123{\bar{D}}_{\mathrm{HJ}}+0.1739$
0.271	2	(*EA*)*_HJ_*	$1191.20{\bar{D}}_{\mathrm{HJ}}-258.80$
		(*EI*)*_HJ_*	$141.78{\bar{D}}_{\mathrm{HJ}}^{3}-43.594{\bar{D}}_{\mathrm{HJ}}^{2}-1.4904{\bar{D}}_{\mathrm{HJ}}+1.6835$
		(*GJ*)*_HJ_*	$132.42{\bar{D}}_{\mathrm{HJ}}^{3}+0.5278{\bar{D}}_{\mathrm{HJ}}^{2}-16.258{\bar{D}}_{\mathrm{HJ}}+2.069$
0.407	3	(*EA*)*_HJ_*	$1261.30{\bar{D}}_{\mathrm{HJ}}-536.11$
		(*EI*)*_HJ_*	$142.83{\bar{D}}_{\mathrm{HJ}}^{3}-67.264{\bar{D}}_{\mathrm{HJ}}^{2}-0.0371{\bar{D}}_{\mathrm{HJ}}+3.4292$
		(*GJ*)*_HJ_*	$132.48{\bar{D}}_{\mathrm{HJ}}^{3}+2.2911{\bar{D}}_{\mathrm{HJ}}^{2}-40.035{\bar{D}}_{\mathrm{HJ}}+9.3192$
0.543	4	(*EA*)*_HJ_*	$1324.10{\bar{D}}_{\mathrm{HJ}}-819.03$
		(*EI*)*_HJ_*	$144.07{\bar{D}}_{\mathrm{HJ}}^{3}-92.596{\bar{D}}_{\mathrm{HJ}}^{2}+4.2067{\bar{D}}_{\mathrm{HJ}}+5.9603$
		(*GJ*)*_HJ_*	$132.49{\bar{D}}_{\mathrm{HJ}}^{3}+4.565{\bar{D}}_{\mathrm{HJ}}^{2}-74.054{\bar{D}}_{\mathrm{HJ}}+23.571$
0.678	5	(*EA*)*_HJ_*	$1320.20{\bar{D}}_{\mathrm{HJ}}-992.12$
		(*EI*)*_HJ_*	$145.65{\bar{D}}_{\mathrm{HJ}}^{3}-120.93{\bar{D}}_{\mathrm{HJ}}^{2}+15.301{\bar{D}}_{\mathrm{HJ}}+6.5399$
		(*GJ*)*_HJ_*	$133.16{\bar{D}}_{\mathrm{HJ}}^{3}+3.0259{\bar{D}}_{\mathrm{HJ}}^{2}-110.4{\bar{D}}_{\mathrm{HJ}}+42.573$
0.814	6	(*EA*)*_HJ_*	$1308.70{\bar{D}}_{\mathrm{HJ}}-1154.20$
		(*EI*)*_HJ_*	$146.64{\bar{D}}_{\mathrm{HJ}}^{3}-146.98{\bar{D}}_{\mathrm{HJ}}^{2}+24.574{\bar{D}}_{\mathrm{HJ}}+9.6188$
		(*GJ*)*_HJ_*	$133.68{\bar{D}}_{\mathrm{HJ}}^{3}+1.6866{\bar{D}}_{\mathrm{HJ}}^{2}-154.64{\bar{D}}_{\mathrm{HJ}}+70.3$
1.086	8	(*EA*)*_HJ_*	$193.15{\bar{D}}_{\mathrm{HJ}}^{2}+403.44{\bar{D}}_{\mathrm{HJ}}-611.86$
		(*EI*)*_HJ_*	$150.15{\bar{D}}_{\mathrm{HJ}}^{3}-210.42{\bar{D}}_{\mathrm{HJ}}^{2}+69.697{\bar{D}}_{\mathrm{HJ}}+6.9541$
		(*GJ*)*_HJ_*	$136.92{\bar{D}}_{\mathrm{HJ}}^{3}-15.923{\bar{D}}_{\mathrm{HJ}}^{2}-238.41{\bar{D}}_{\mathrm{HJ}}+143.84$
1.221	9	(*EA*)*_HJ_*	$180.10{\bar{D}}_{\mathrm{HJ}}^{2}+339.22{\bar{D}}_{\mathrm{HJ}}-583.23$
		(*EI*)*_HJ_*	$151.24{\bar{D}}_{\mathrm{HJ}}^{3}-240.15{\bar{D}}_{\mathrm{HJ}}^{2}+94.725{\bar{D}}_{\mathrm{HJ}}+4.6415$
		(*GJ*)*_HJ_*	$138.41{\bar{D}}_{\mathrm{HJ}}^{3}-28.583{\bar{D}}_{\mathrm{HJ}}^{2}-273.47{\bar{D}}_{\mathrm{HJ}}+181.06$
1.357	10	(*EA*)*_HJ_*	$284.23{\bar{D}}_{\mathrm{HJ}}^{2}-186.58{\bar{D}}_{\mathrm{HJ}}-147.44$
		(*EI*)*_HJ_*	$154.04{\bar{D}}_{\mathrm{HJ}}^{3}-281.32{\bar{D}}_{\mathrm{HJ}}^{2}+144.68{\bar{D}}_{\mathrm{HJ}}-10.904$
		(*GJ*)*_HJ_*	$144.83{\bar{D}}_{\mathrm{HJ}}^{3}-73.238{\bar{D}}_{\mathrm{HJ}}^{2}-251.68{\bar{D}}_{\mathrm{HJ}}+193.39$
1.628	12	(*EA*)*_HJ_*	$296.06{\bar{D}}_{\mathrm{HJ}}^{2}-449.51{\bar{D}}_{\mathrm{HJ}}+87.69$
		(*EI*)*_HJ_*	$156.99{\bar{D}}_{\mathrm{HJ}}^{3}-349.56{\bar{D}}_{\mathrm{HJ}}^{2}+228.31{\bar{D}}_{\mathrm{HJ}}-30.945$
		(*GJ*)*_HJ_*	$149.77{\bar{D}}_{\mathrm{HJ}}^{3}-118.45{\bar{D}}_{\mathrm{HJ}}^{2}-298.53{\bar{D}}_{\mathrm{HJ}}+291.77$
2.035	15	(*EA*)*_HJ_*	$302.53{\bar{D}}_{\mathrm{HJ}}^{2}-783.21{\bar{D}}_{\mathrm{HJ}}+505.78$
		(*EI*)*_HJ_*	$162.58{\bar{D}}_{\mathrm{HJ}}^{3}-467.74{\bar{D}}_{\mathrm{HJ}}^{2}+423.39{\bar{D}}_{\mathrm{HJ}}-111.88$
		(*GJ*)*_HJ_*	$166.3{\bar{D}}_{\mathrm{HJ}}^{3}-276.92{\bar{D}}_{\mathrm{HJ}}^{2}-133.16{\bar{D}}_{\mathrm{HJ}}+291.12$

The R-square value is always better than *R*^2^ = 0.9972, for (*EA*)*_HJ_*, and better than *R*^2^ = 0.9999, for (*EI*)*_HJ_* and (*GJ*)*_HJ_*.

**Table A3:** Equations fitted to the results in Figure 10b, Figure 11b, and Figure 12b, for the cases of the tensile, (*EA*)*_HJ_*, bending, (*EI*)*_HJ_*, and torsional, (*GJ*)*_HJ_* rigidities of zigzag–zigzag HJs.

Δ*D*, nm	*C*	Rigid.	Fitting Equations
0.078	1	(*EA*)*_HJ_*	$1134{\bar{D}}_{\mathrm{HJ}}-42.291$
		(*EI*)*_HJ_*	$138.84{\bar{D}}_{\mathrm{HJ}}^{3}-12.253{\bar{D}}_{\mathrm{HJ}}^{2}-0.1733{\bar{D}}_{\mathrm{HJ}}+0.0506$
		(*GJ*)*_HJ_*	$132.58{\bar{D}}_{\mathrm{HJ}}^{3}+0.029{\bar{D}}_{\mathrm{HJ}}^{2}-1.269{\bar{D}}_{\mathrm{HJ}}+0.0315$
0.157	2	(*EA*)*_HJ_*	$1182.1{\bar{D}}_{\mathrm{HJ}}-148.89$
		(*EI*)*_HJ_*	$139.35{\bar{D}}_{\mathrm{HJ}}^{3}-24.737{\bar{D}}_{\mathrm{HJ}}^{2}-0.4883{\bar{D}}_{\mathrm{HJ}}+0.3184$
		(*GJ*)*_HJ_*	$132.68{\bar{D}}_{\mathrm{HJ}}^{3}+0.3053{\bar{D}}_{\mathrm{HJ}}^{2}-5.4299{\bar{D}}_{\mathrm{HJ}}+0.399$
0.235	3	(*EA*)*_HJ_*	$1250.5{\bar{D}}_{\mathrm{HJ}}-307.65$
		(*EI*)*_HJ_*	$140.03{\bar{D}}_{\mathrm{HJ}}^{3}-38.073{\bar{D}}_{\mathrm{HJ}}^{2}-0.0121{\bar{D}}_{\mathrm{HJ}}+0.647$
		(*GJ*)*_HJ_*	$132.62{\bar{D}}_{\mathrm{HJ}}^{3}+1.3242{\bar{D}}_{\mathrm{HJ}}^{2}-13.36{\bar{D}}_{\mathrm{HJ}}+1.7955$
0.313	4	(*EA*)*_HJ_*	$1311.5{\bar{D}}_{\mathrm{HJ}}-429.26$
		(*EI*)*_HJ_*	$140.9{\bar{D}}_{\mathrm{HJ}}^{3}-52.282{\bar{D}}_{\mathrm{HJ}}^{2}+1.3713{\bar{D}}_{\mathrm{HJ}}+1.1218$
		(*GJ*)*_HJ_*	$132.53{\bar{D}}_{\mathrm{HJ}}^{3}+2.6363{\bar{D}}_{\mathrm{HJ}}^{2}-24.692{\bar{D}}_{\mathrm{HJ}}+4.5375$
0.392	5	(*EA*)*_HJ_*	$1306.1{\bar{D}}_{\mathrm{HJ}}-567.43$
		(*EI*)*_HJ_*	$142.09{\bar{D}}_{\mathrm{HJ}}^{3}-68.109{\bar{D}}_{\mathrm{HJ}}^{2}+4.9756{\bar{D}}_{\mathrm{HJ}}+1.2278$
		(*GJ*)*_HJ_*	$133.09{\bar{D}}_{\mathrm{HJ}}^{3}+1.746{\bar{D}}_{\mathrm{HJ}}^{2}-36.78{\bar{D}}_{\mathrm{HJ}}+8.1887$
0.470	6	(*EA*)*_HJ_*	$1293.6{\bar{D}}_{\mathrm{HJ}}-659.51$
		(*EI*)*_HJ_*	$142.7{\bar{D}}_{\mathrm{HJ}}^{3}-82.58{\bar{D}}_{\mathrm{HJ}}^{2}+7.9715{\bar{D}}_{\mathrm{HJ}}+1.8014$
		(*GJ*)*_HJ_*	$133.5{\bar{D}}_{\mathrm{HJ}}^{3}+0.9724{\bar{D}}_{\mathrm{HJ}}^{2}-51.475{\bar{D}}_{\mathrm{HJ}}+13.511$
0.627	8	(*EA*)*_HJ_*	$332.04{\bar{D}}_{\mathrm{HJ}}^{2}+392.23{\bar{D}}_{\mathrm{HJ}}-345.88$
		(*EI*)*_HJ_*	$145.4{\bar{D}}_{\mathrm{HJ}}^{3}-117.64{\bar{D}}_{\mathrm{HJ}}^{2}+22.49{\bar{D}}_{\mathrm{HJ}}+1.296$
		(*GJ*)*_HJ_*	$136.51{\bar{D}}_{\mathrm{HJ}}^{3}-9.1658{\bar{D}}_{\mathrm{HJ}}^{2}-79.233{\bar{D}}_{\mathrm{HJ}}+27.599$
0.705	9	(*EA*)*_HJ_*	$309.06{\bar{D}}_{\mathrm{HJ}}^{2}+329.24{\bar{D}}_{\mathrm{HJ}}-329.43$
		(*EI*)*_HJ_*	$146.1{\bar{D}}_{\mathrm{HJ}}^{3}-133.94{\bar{D}}_{\mathrm{HJ}}^{2}+30.502{\bar{D}}_{\mathrm{HJ}}+0.8629$
		(*GJ*)*_HJ_*	$137.88{\bar{D}}_{\mathrm{HJ}}^{3}-16.439{\bar{D}}_{\mathrm{HJ}}^{2}-90.809{\bar{D}}_{\mathrm{HJ}}+34.713$
0.783	10	(*EA*)*_HJ_*	$332.04{\bar{D}}_{\mathrm{HJ}}^{2}+392.23{\bar{D}}_{\mathrm{HJ}}-345.88$
		(*EI*)*_HJ_*	$148.44{\bar{D}}_{\mathrm{HJ}}^{3}-156.52{\bar{D}}_{\mathrm{HJ}}^{2}+46.476{\bar{D}}_{\mathrm{HJ}}-2.0223$
		(*GJ*)*_HJ_*	$144.16{\bar{D}}_{\mathrm{HJ}}^{3}-42.089{\bar{D}}_{\mathrm{HJ}}^{2}-83.507{\bar{D}}_{\mathrm{HJ}}+37.045$
0.940	12	(*EA*)*_HJ_*	$504.89{\bar{D}}_{\mathrm{HJ}}^{2}-445.72{\bar{D}}_{\mathrm{HJ}}+52.25$
		(*EI*)*_HJ_*	$150.55{\bar{D}}_{\mathrm{HJ}}^{3}-193.55{\bar{D}}_{\mathrm{HJ}}^{2}+72.982{\bar{D}}_{\mathrm{HJ}}-5.7112$
		(*GJ*)*_HJ_*	$148.83{\bar{D}}_{\mathrm{HJ}}^{3}-67.964{\bar{D}}_{\mathrm{HJ}}^{2}-98.892{\bar{D}}_{\mathrm{HJ}}+55.802$
1.175	15	(*EA*)*_HJ_*	$513.61{\bar{D}}_{\mathrm{HJ}}^{2}-769.18{\bar{D}}_{\mathrm{HJ}}+287.53$
		(*EI*)*_HJ_*	$154.79{\bar{D}}_{\mathrm{HJ}}^{3}-257.12{\bar{D}}_{\mathrm{HJ}}^{2}+134.37{\bar{D}}_{\mathrm{HJ}}-20.5$
		(*GJ*)*_HJ_*	$164.87{\bar{D}}_{\mathrm{HJ}}^{3}-158.5{\bar{D}}_{\mathrm{HJ}}^{2}-44.003{\bar{D}}_{\mathrm{HJ}}+55.543$

The R-square value is always better than *R*^2^ = 0.9966, for (*EA*)*_HJ_*, and better than *R*^2^ = 0.9999, for (*EI*)*_HJ_* and (*GJ*)*_HJ_*.
